# Supplementary figures and images for: Simulation of 69 microbial communities indicates sequencing depth and false positives are major drivers of bias in prokaryotic metagenome-assembled genome recovery
Source: PLoS Comput Biol. 2024 Oct 22;20(10):e1012530. doi: 10.1371/journal.pcbi.1012530 (PMC11530072; doi:10.1371/journal.pcbi.1012530)

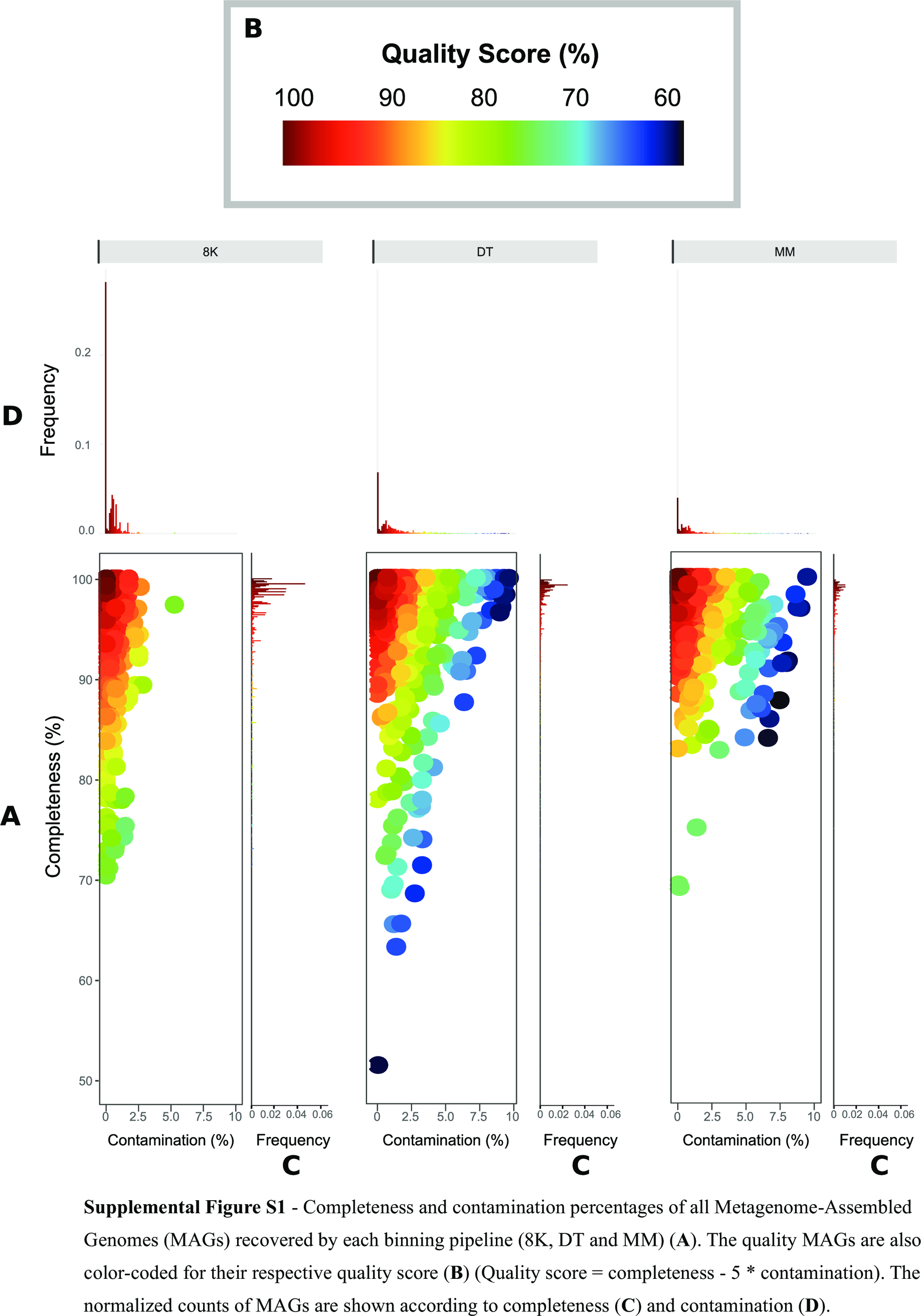

Supplement: S1 Fig — Completeness and contamination percentages of all Metagenome-Assembled Genomes (MAGs) recovered by each binning pipeline (8K DT and MM) (A). The quality MAGs are also color-coded for their respective quality score (B) (Quality score = completeness—5 * contamination). The normalized counts of MAGs are shown according to completeness (C) and contamination (D). (TIF) [file pcbi.1012530.s001.tif]

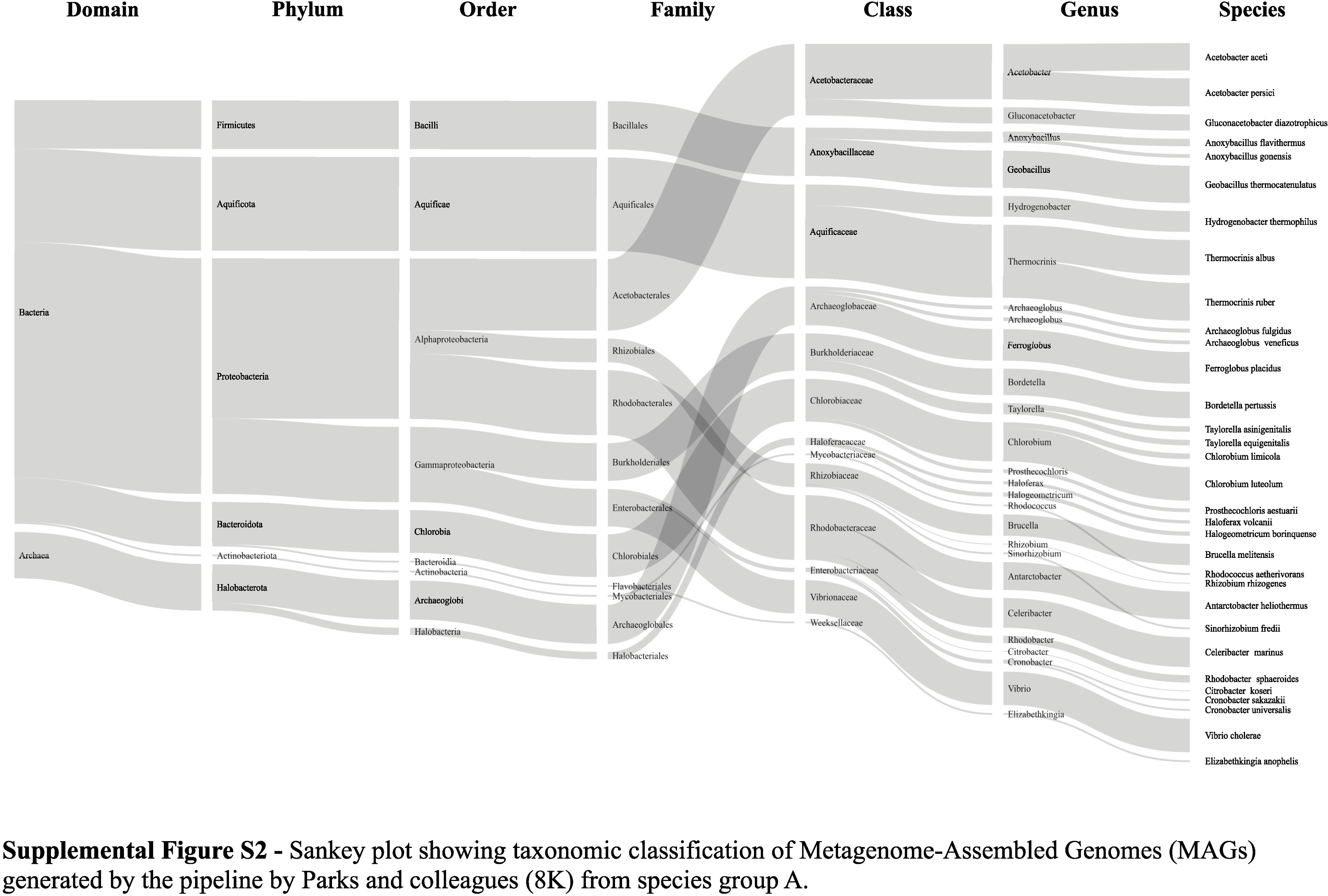

Supplement: S2 Fig — (TIF) [file pcbi.1012530.s002.tif]

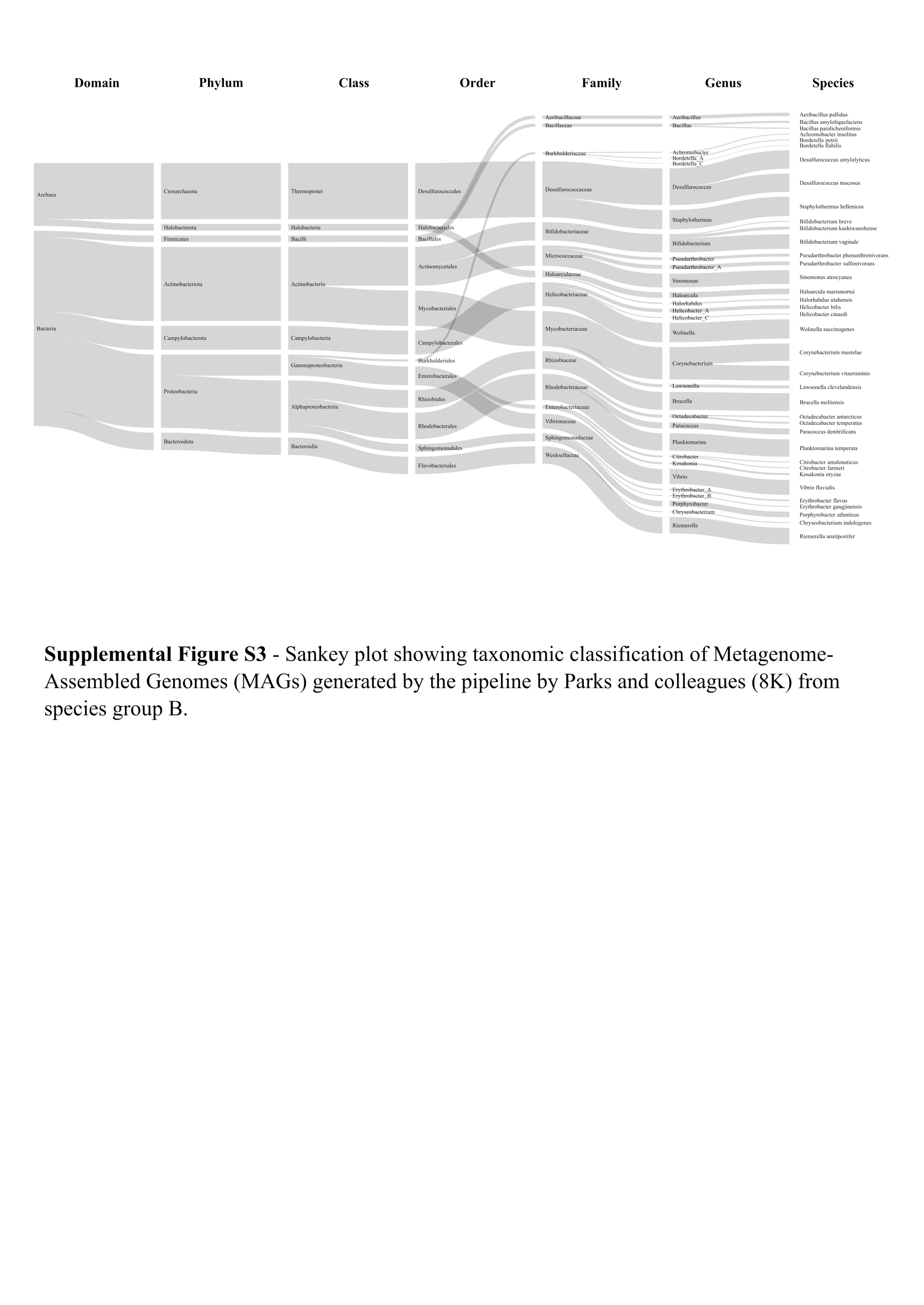

Supplement: S3 Fig — (TIF) [file pcbi.1012530.s003.tif]

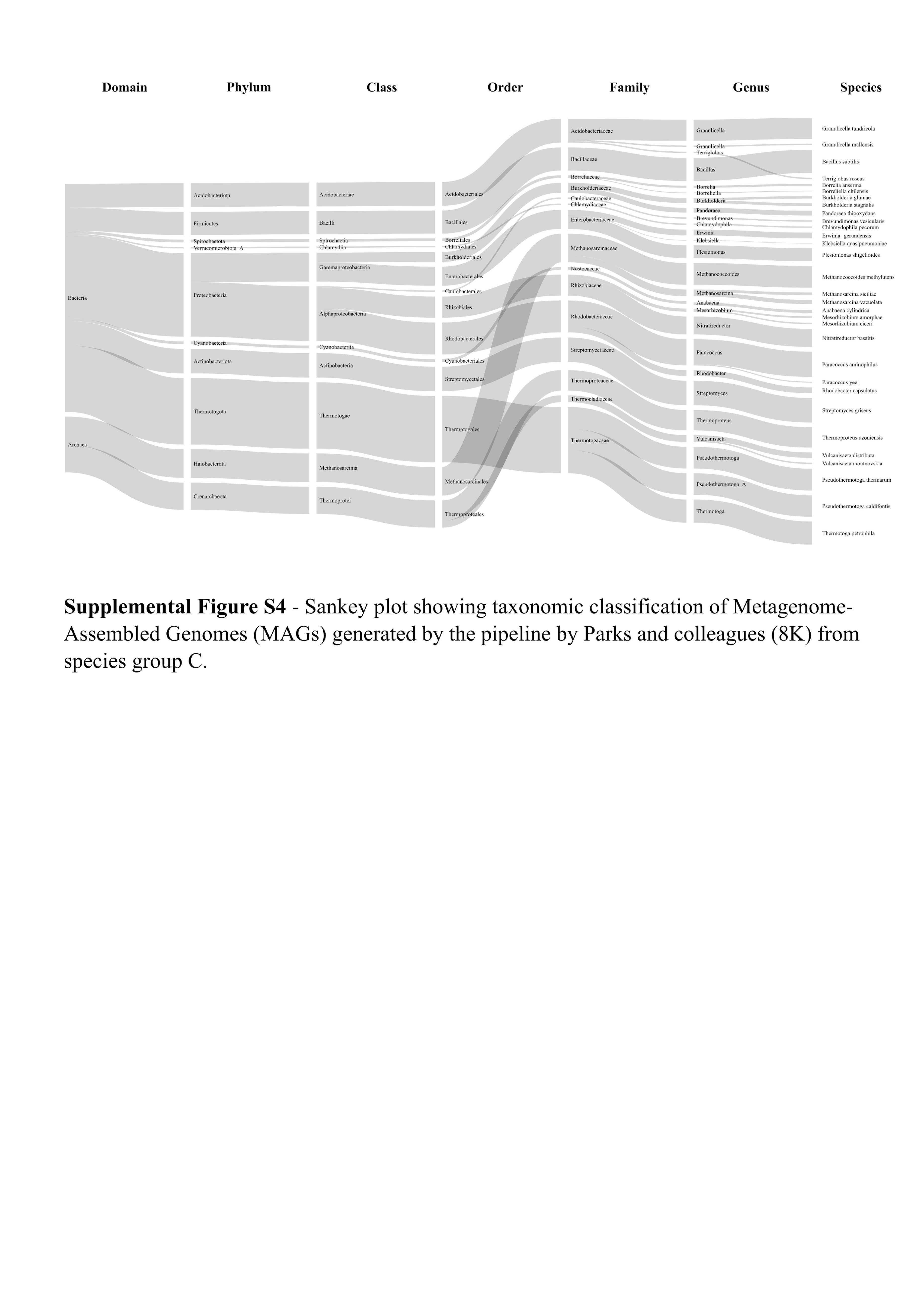

Supplement: S4 Fig — (TIF) [file pcbi.1012530.s004.tif]

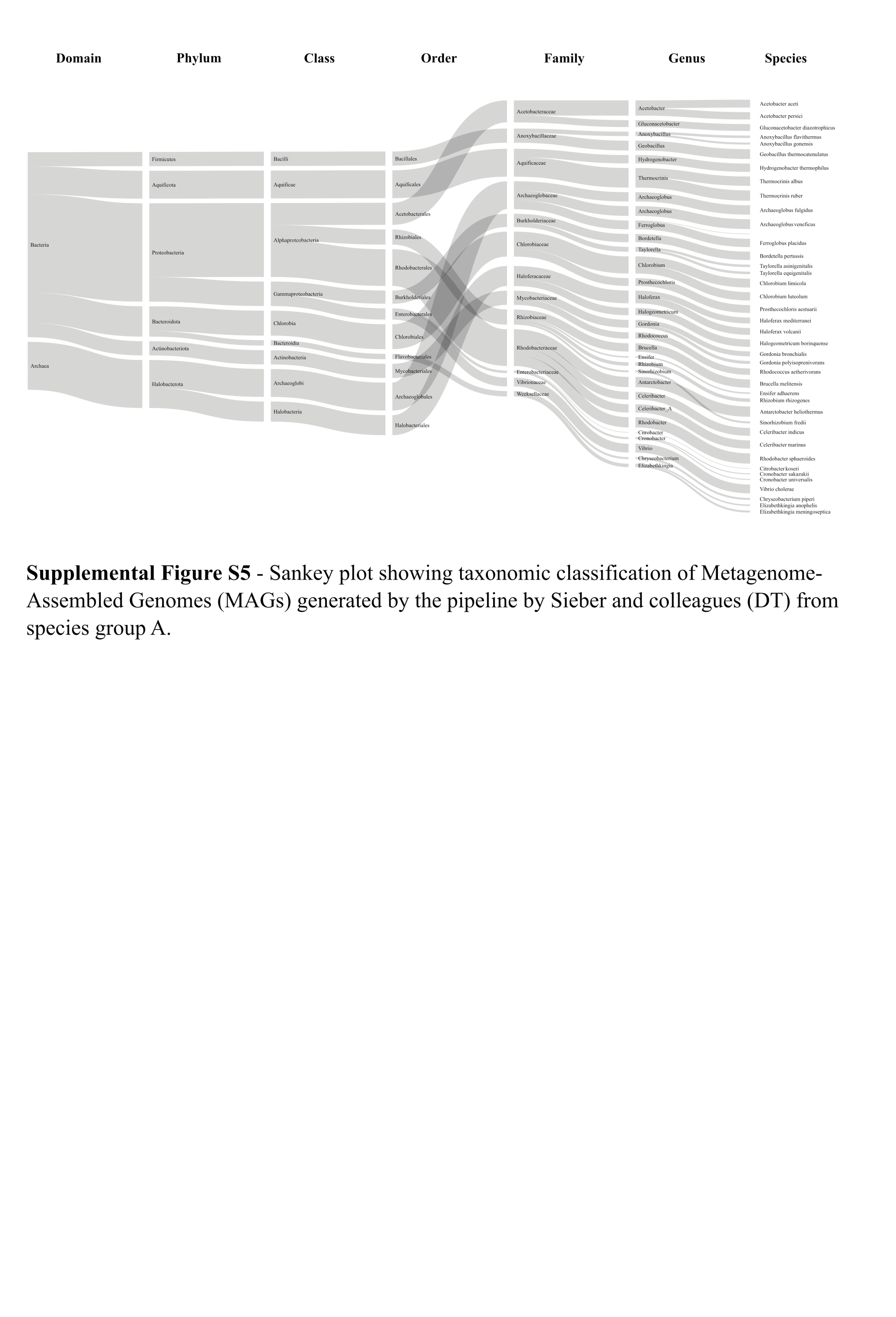

Supplement: S5 Fig — (TIF) [file pcbi.1012530.s005.tif]

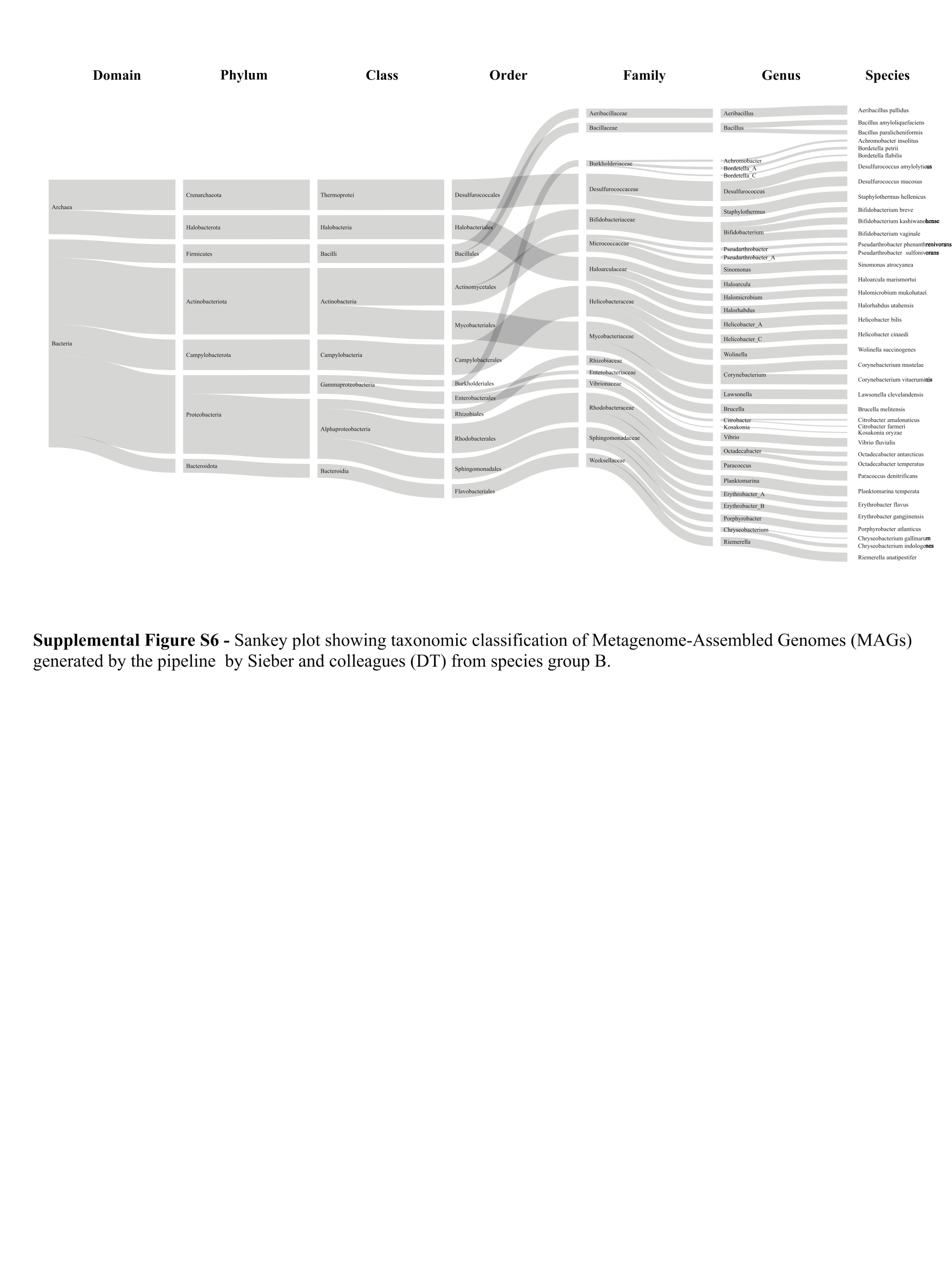

Supplement: S6 Fig — (TIF) [file pcbi.1012530.s006.tif]

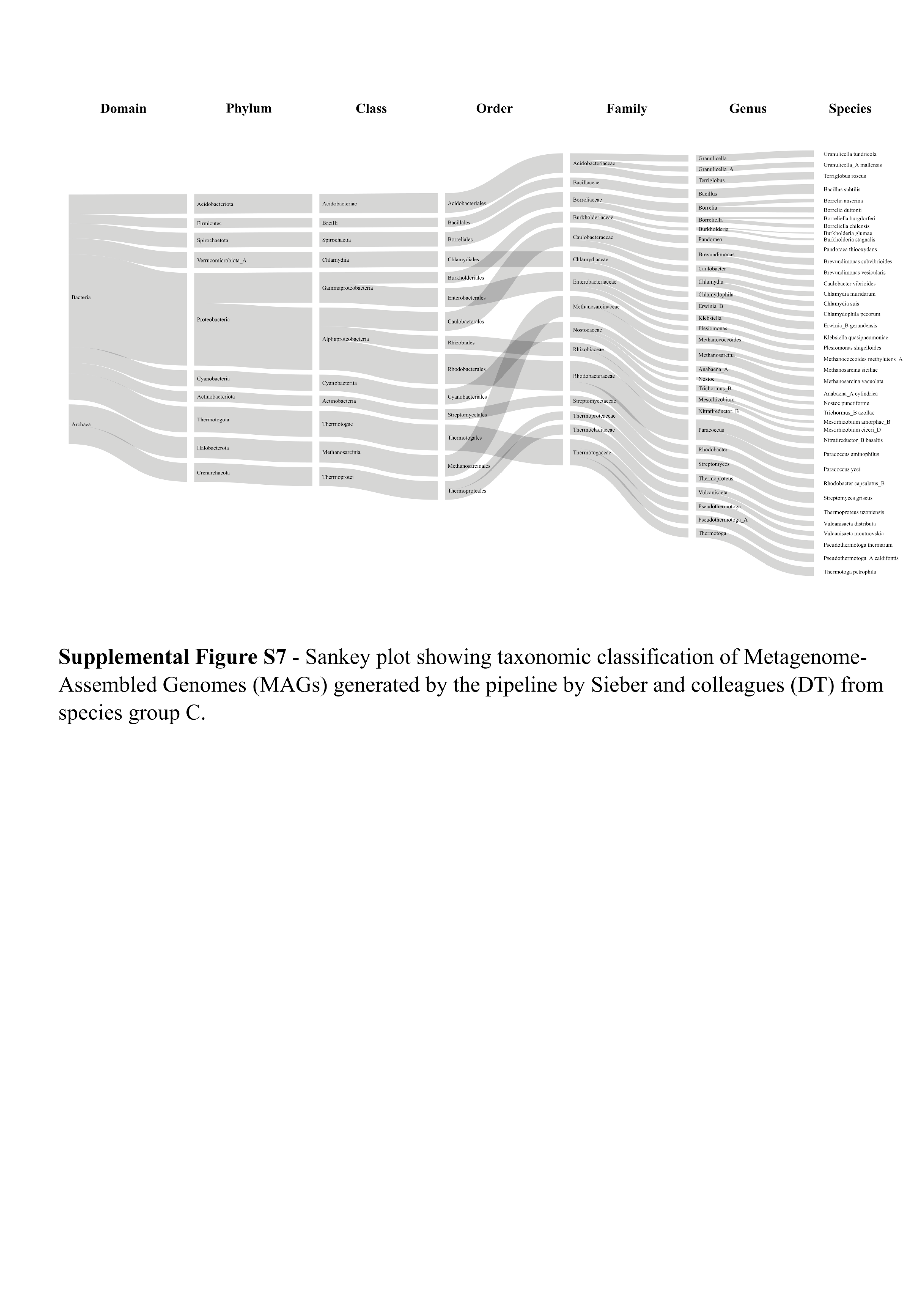

Supplement: S7 Fig — (TIF) [file pcbi.1012530.s007.tif]

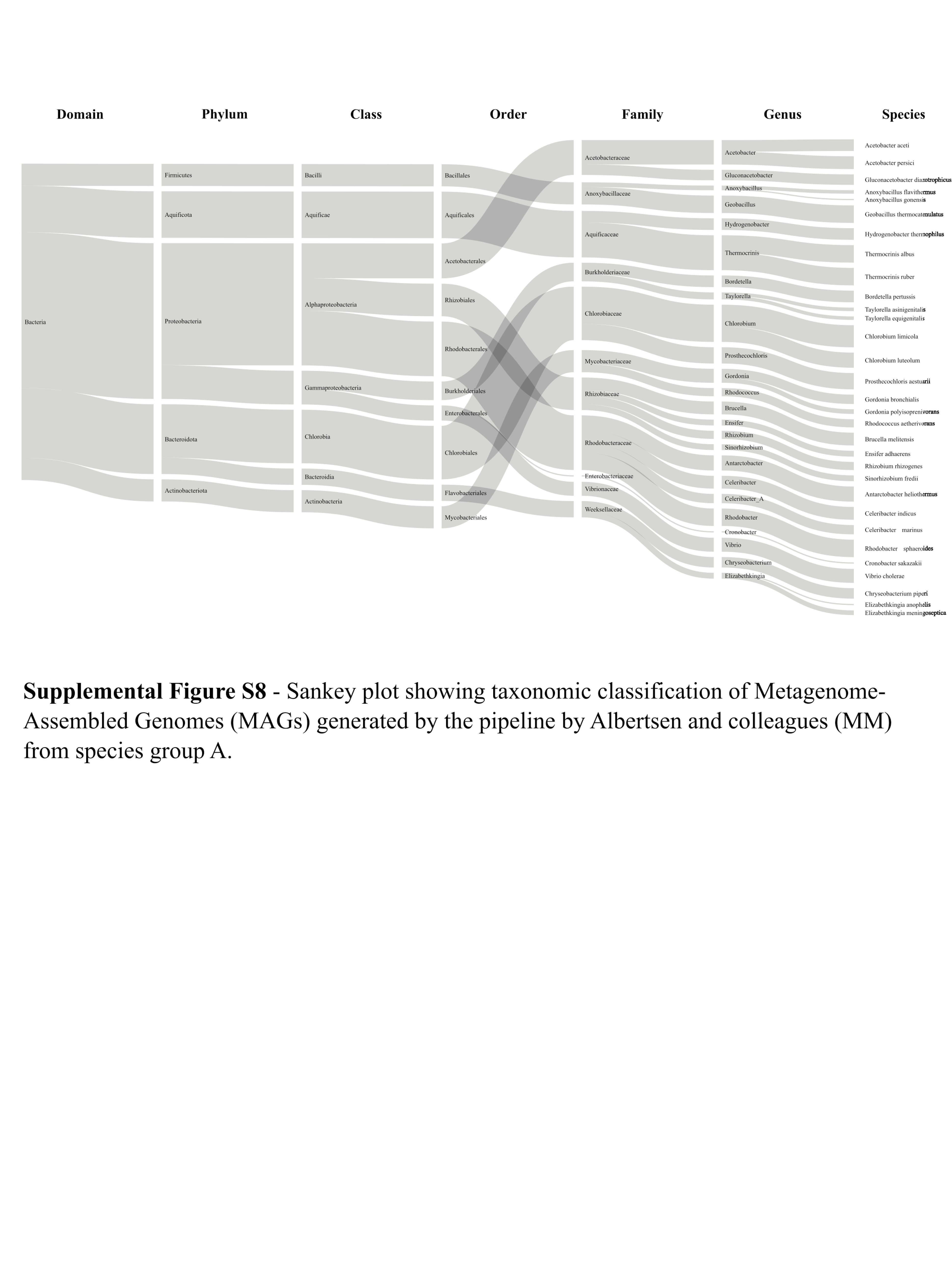

Supplement: S8 Fig — (TIF) [file pcbi.1012530.s008.tif]

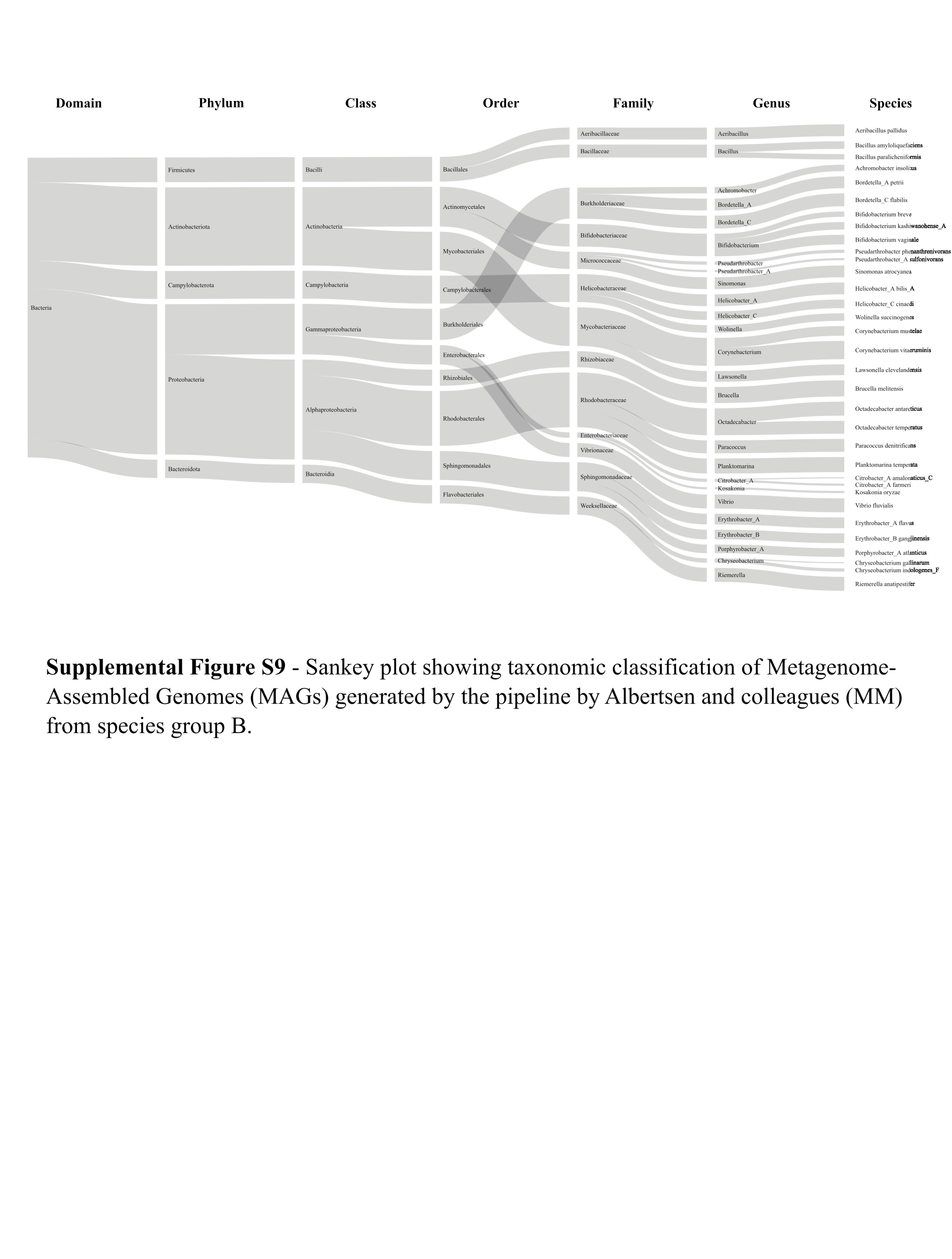

Supplement: S9 Fig — (TIF) [file pcbi.1012530.s009.tif]

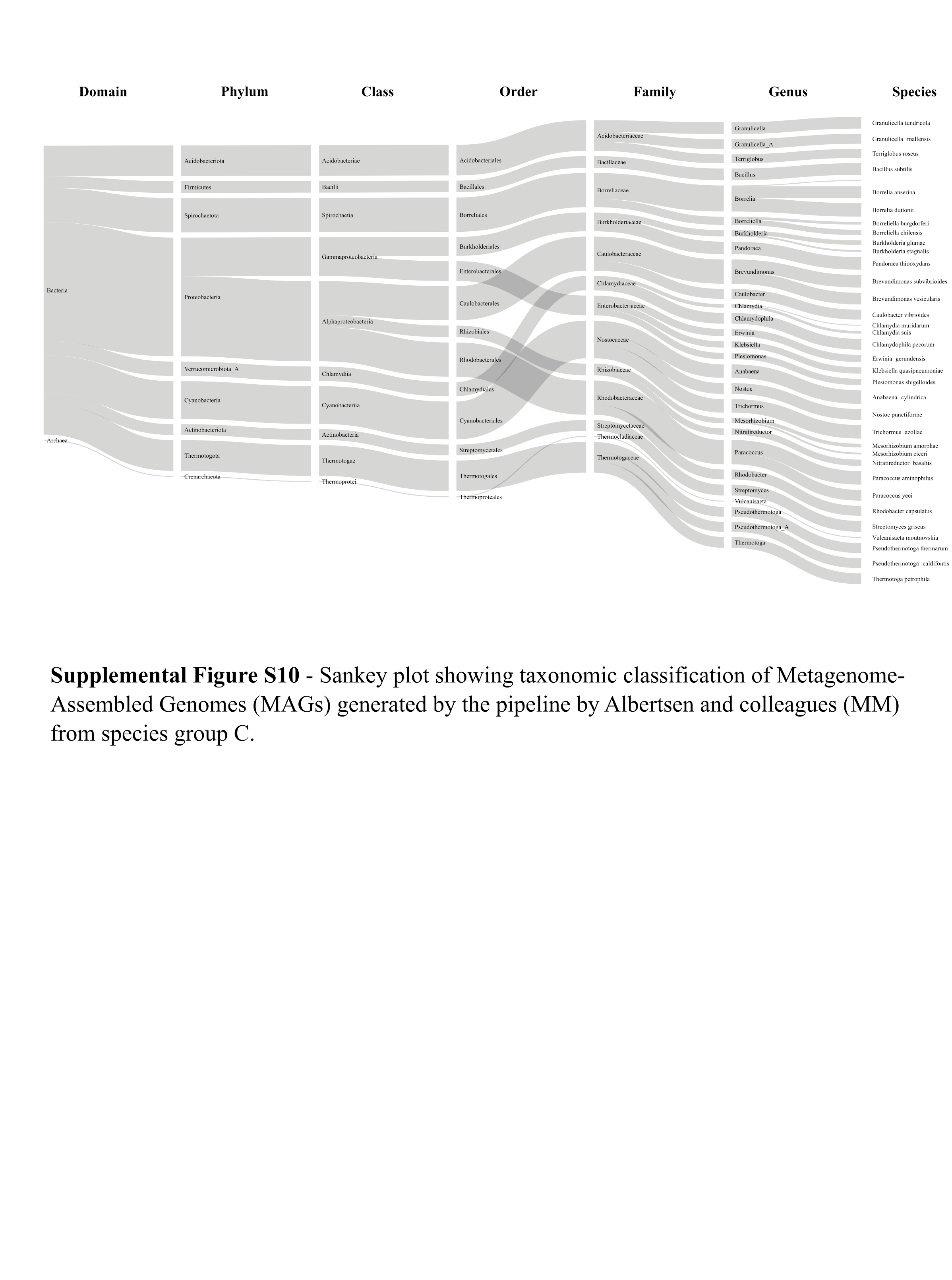

Supplement: S10 Fig — (TIF) [file pcbi.1012530.s010.tif]

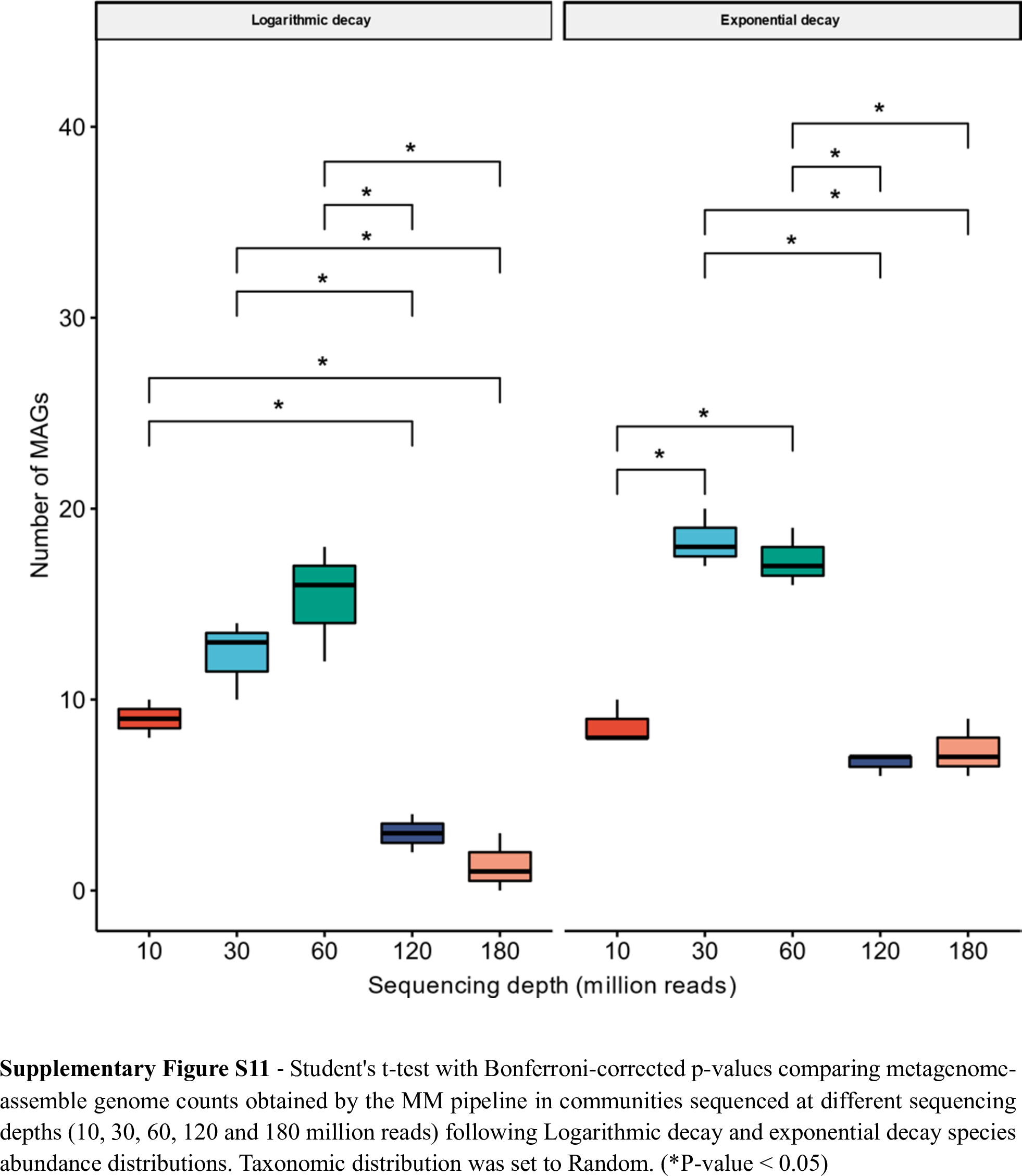

Supplement: S11 Fig — Taxonomic distribution was set to Random. (*P-value < 0.05) (TIF) [file pcbi.1012530.s011.tif]

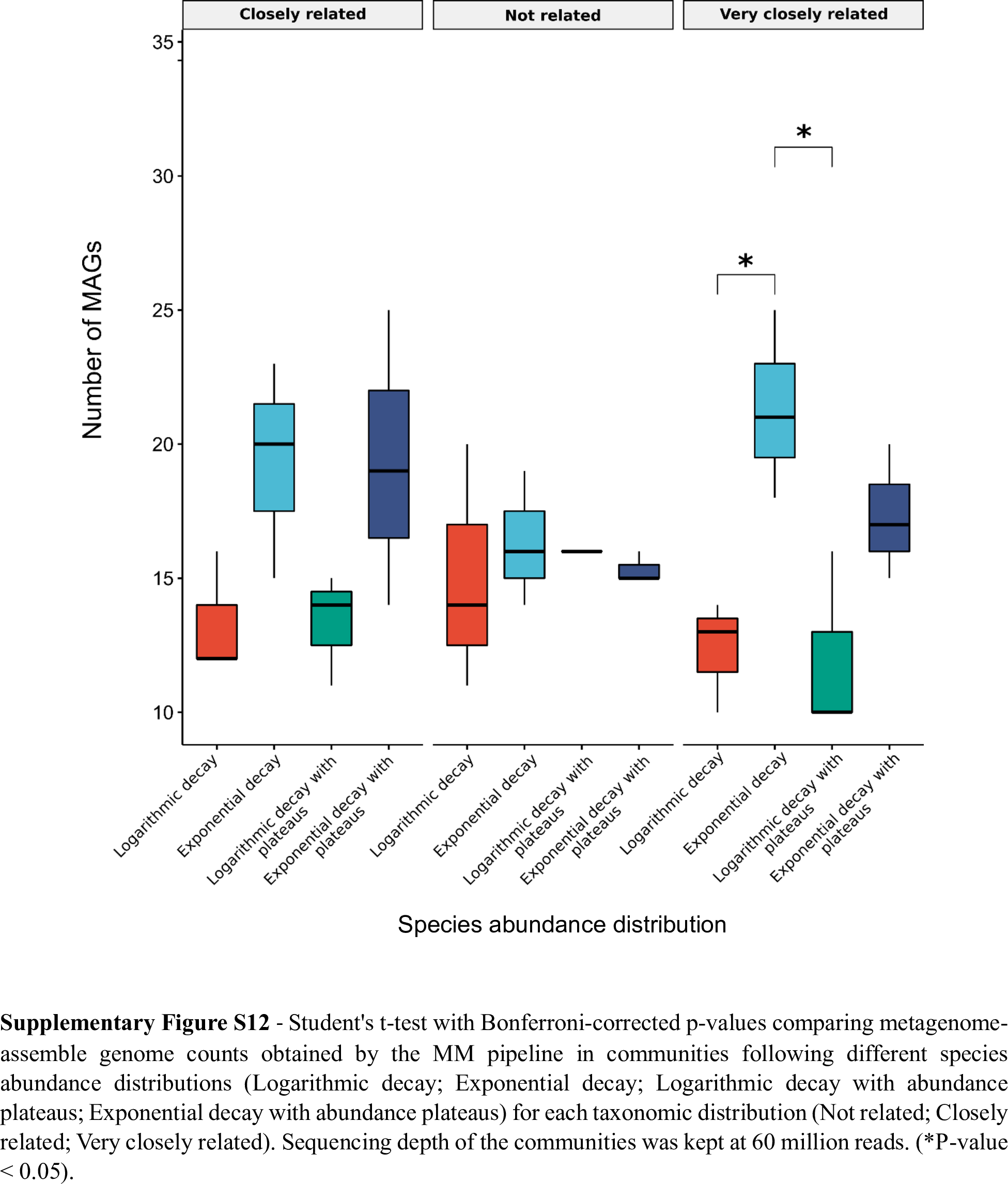

Supplement: S12 Fig — Sequencing depth of the communities was kept at 60 million reads. (*P-value < 0.05) (TIF) [file pcbi.1012530.s012.tif]

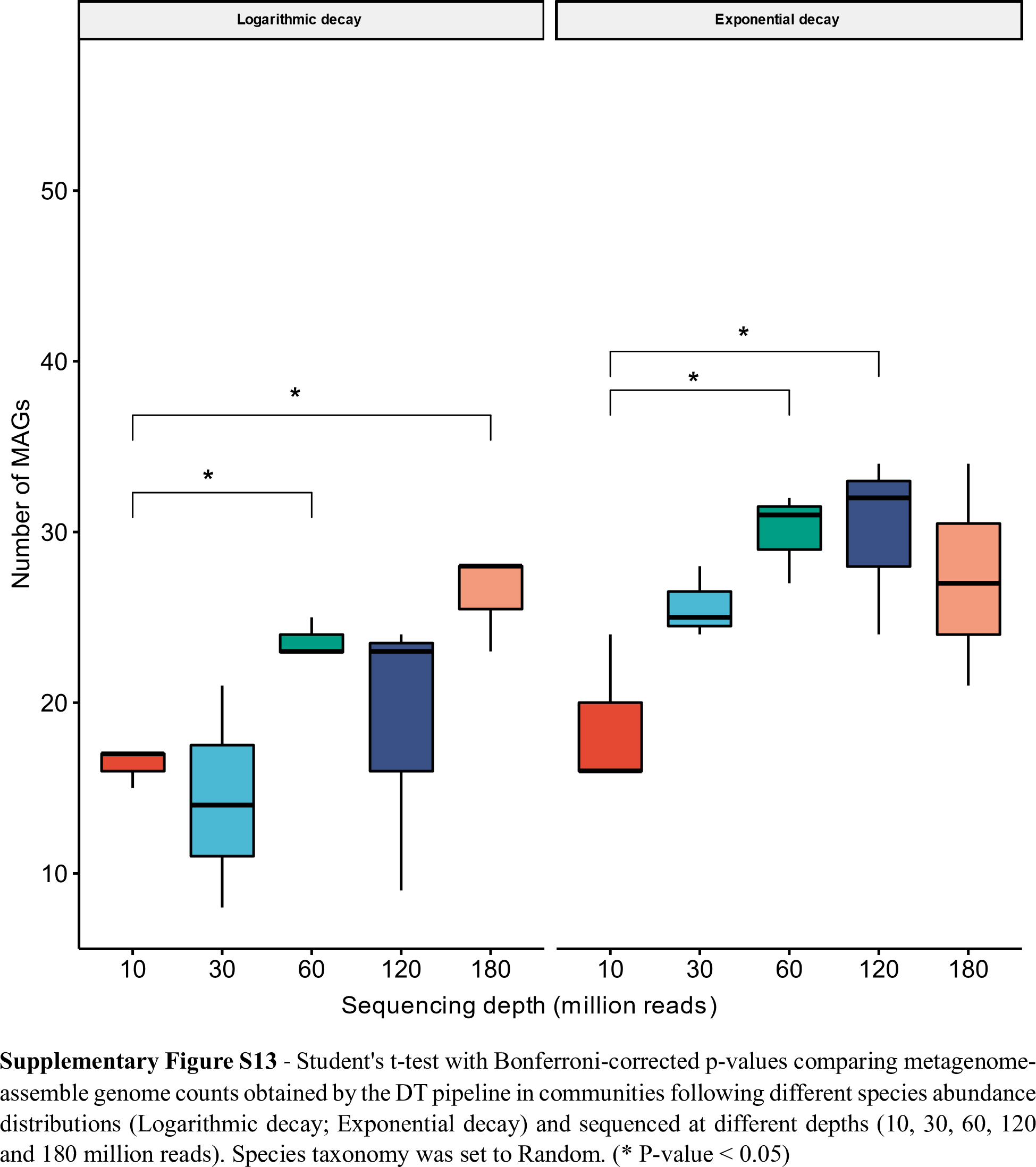

Supplement: S13 Fig — Species taxonomy was set to Random. (* P-value < 0.05) (TIF) [file pcbi.1012530.s013.tif]

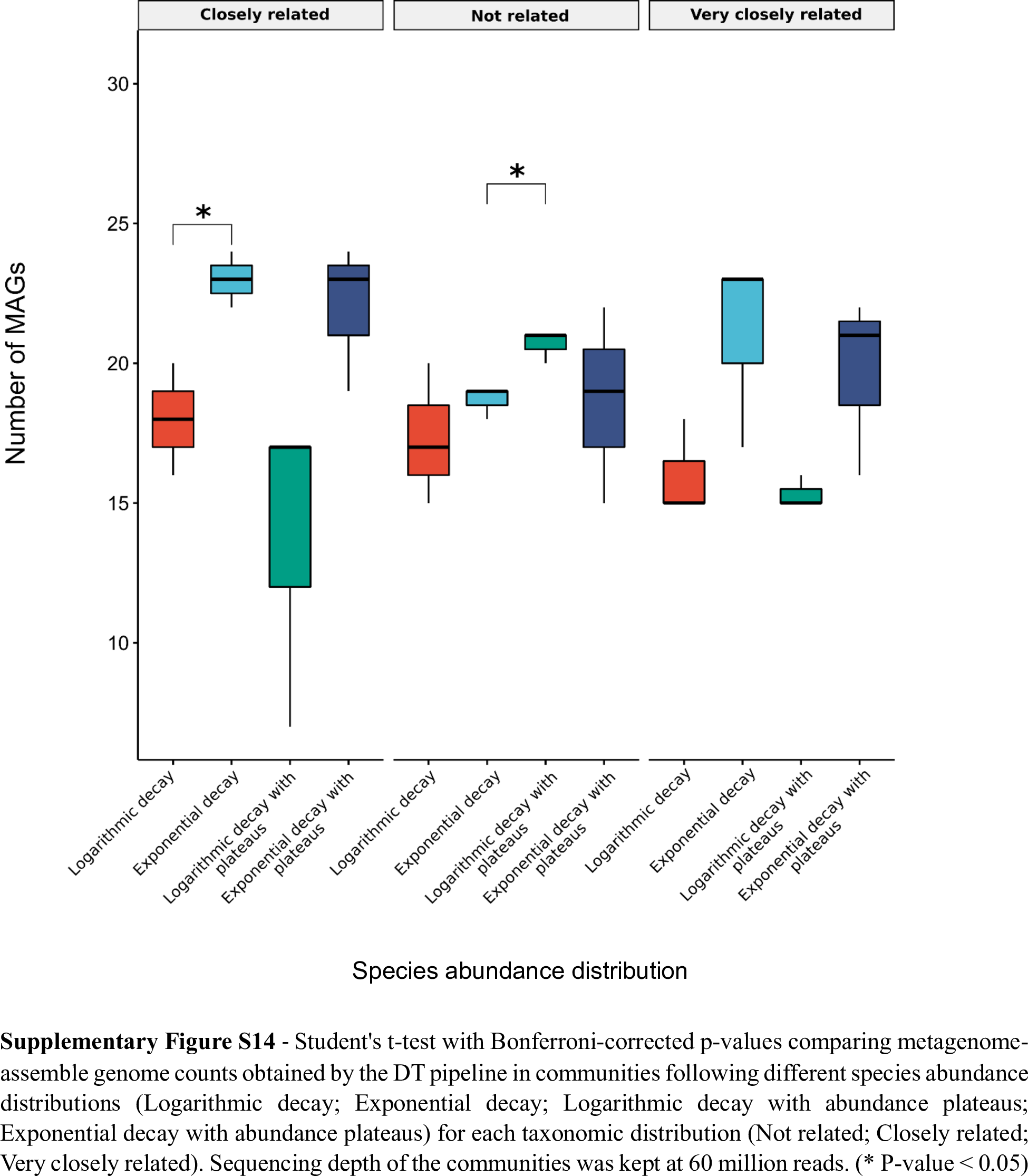

Supplement: S14 Fig — Sequencing depth of the communities was kept at 60 million reads. (* P-value < 0.05) (TIF) [file pcbi.1012530.s014.tif]

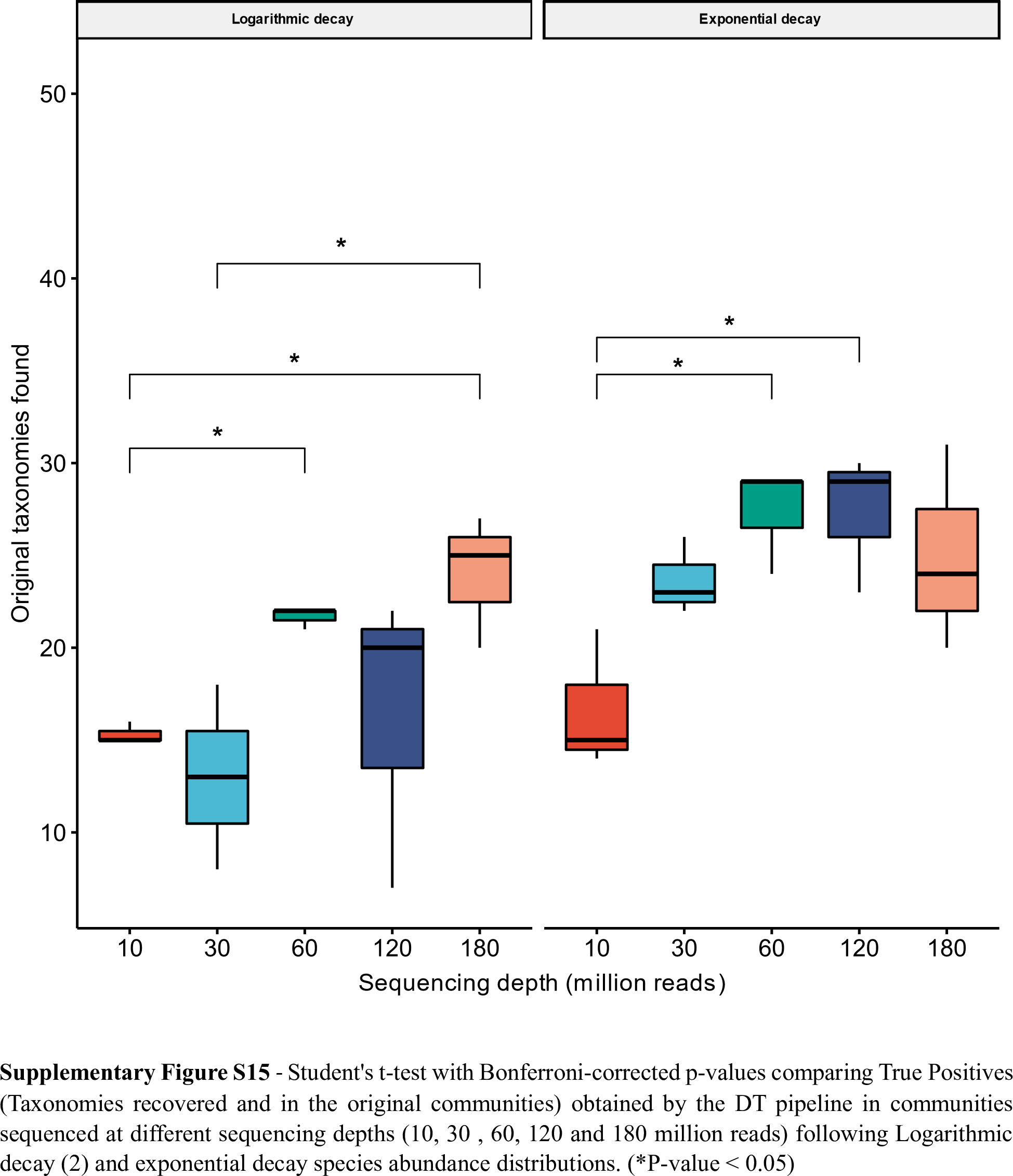

Supplement: S15 Fig — (*P-value < 0.05) (TIF) [file pcbi.1012530.s015.tif]

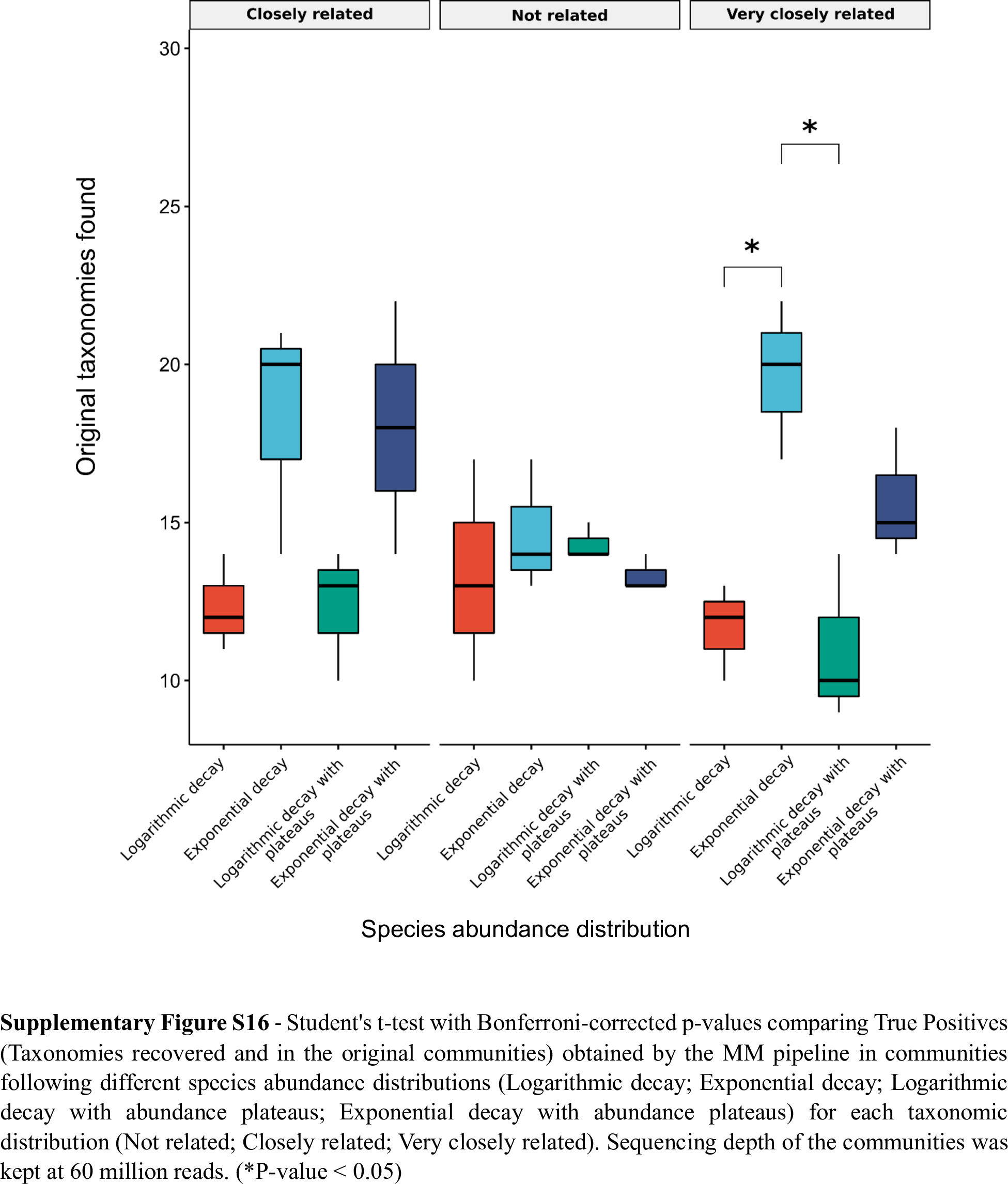

Supplement: S16 Fig — Sequencing depth of the communities was kept at 60 million reads. (*P-value < 0.05) (TIF) [file pcbi.1012530.s016.tif]

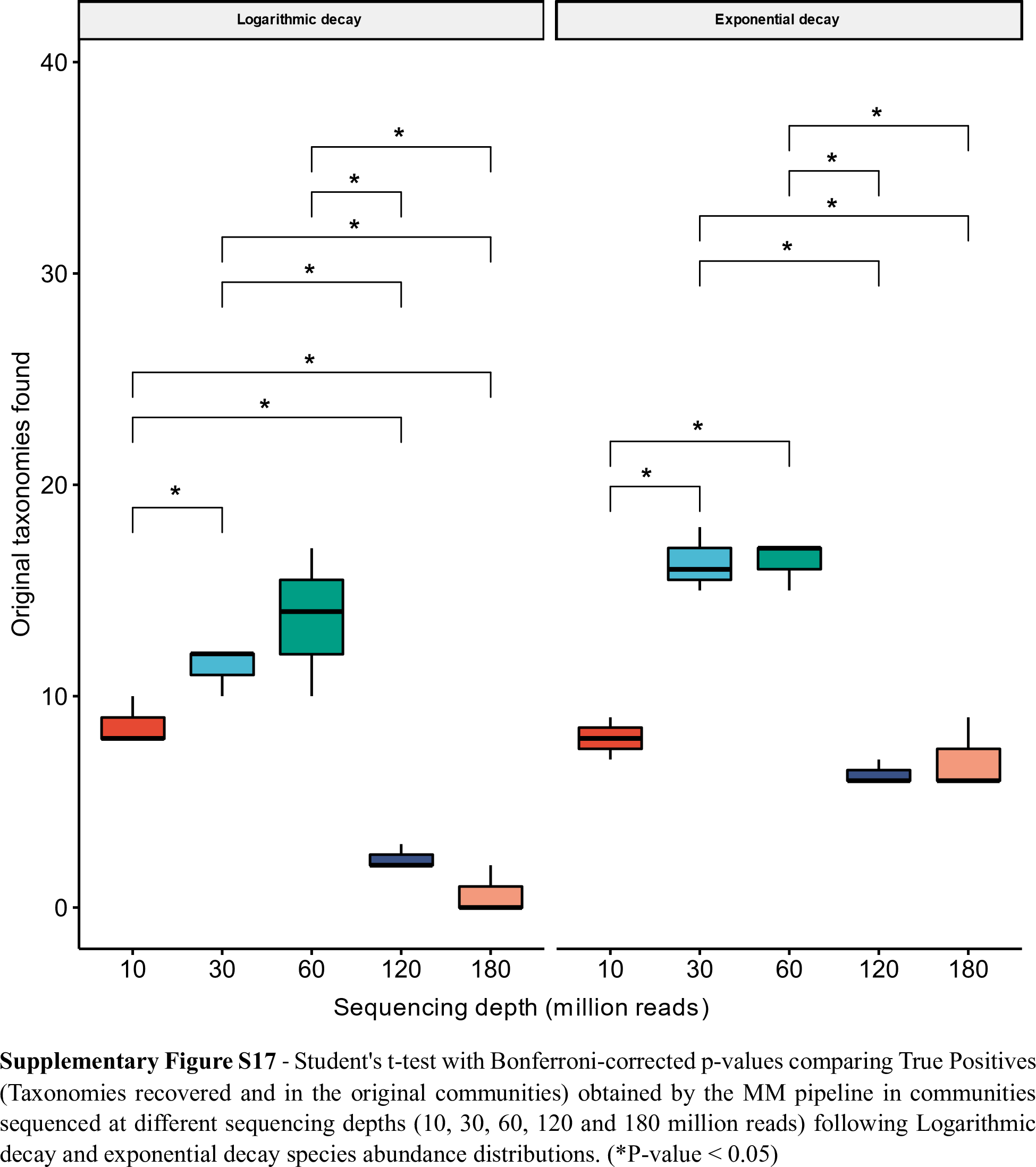

Supplement: S17 Fig — (*P-value < 0.05) (TIF) [file pcbi.1012530.s017.tif]

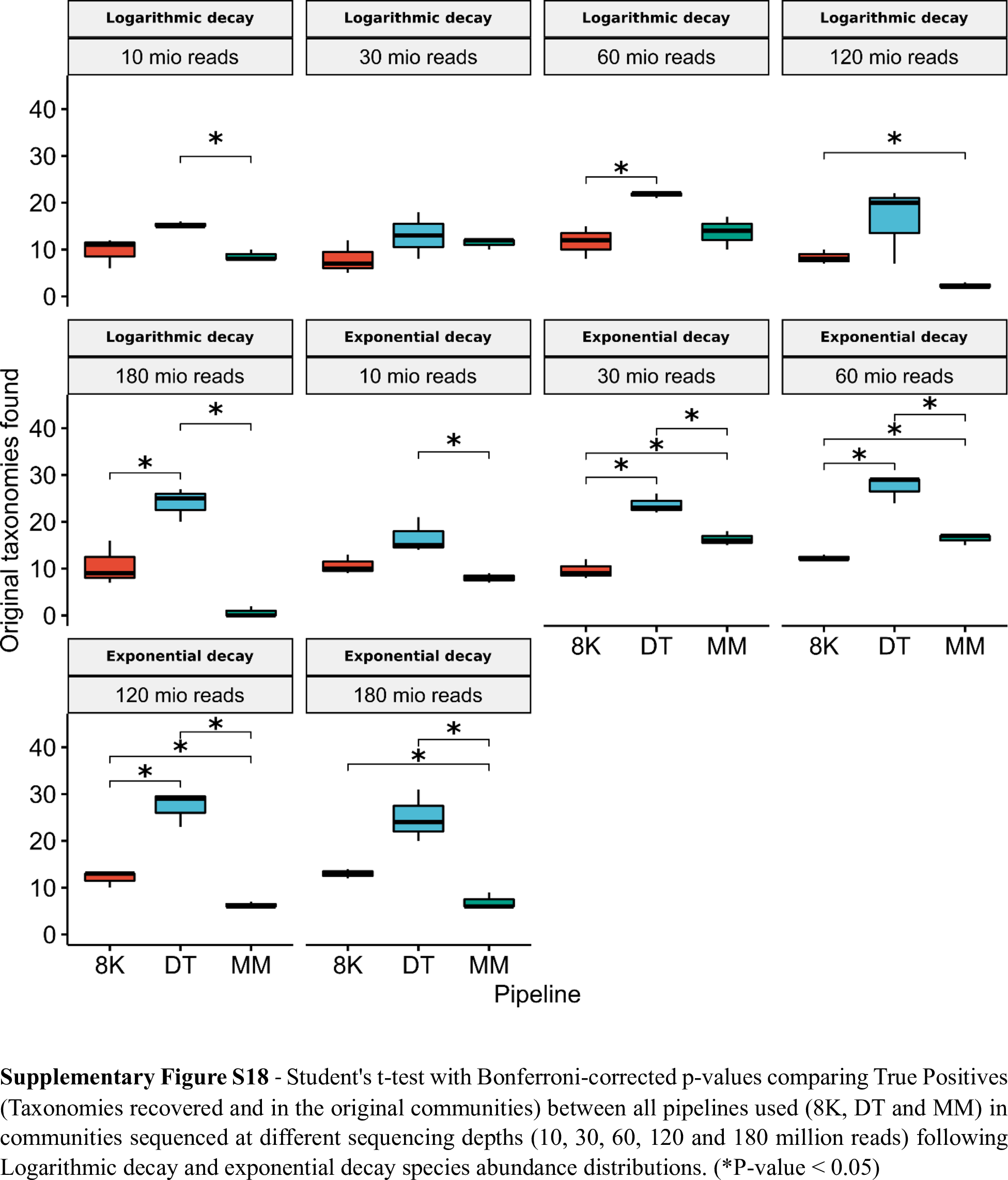

Supplement: S18 Fig — (*P-value < 0.05) (TIF) [file pcbi.1012530.s018.tif]

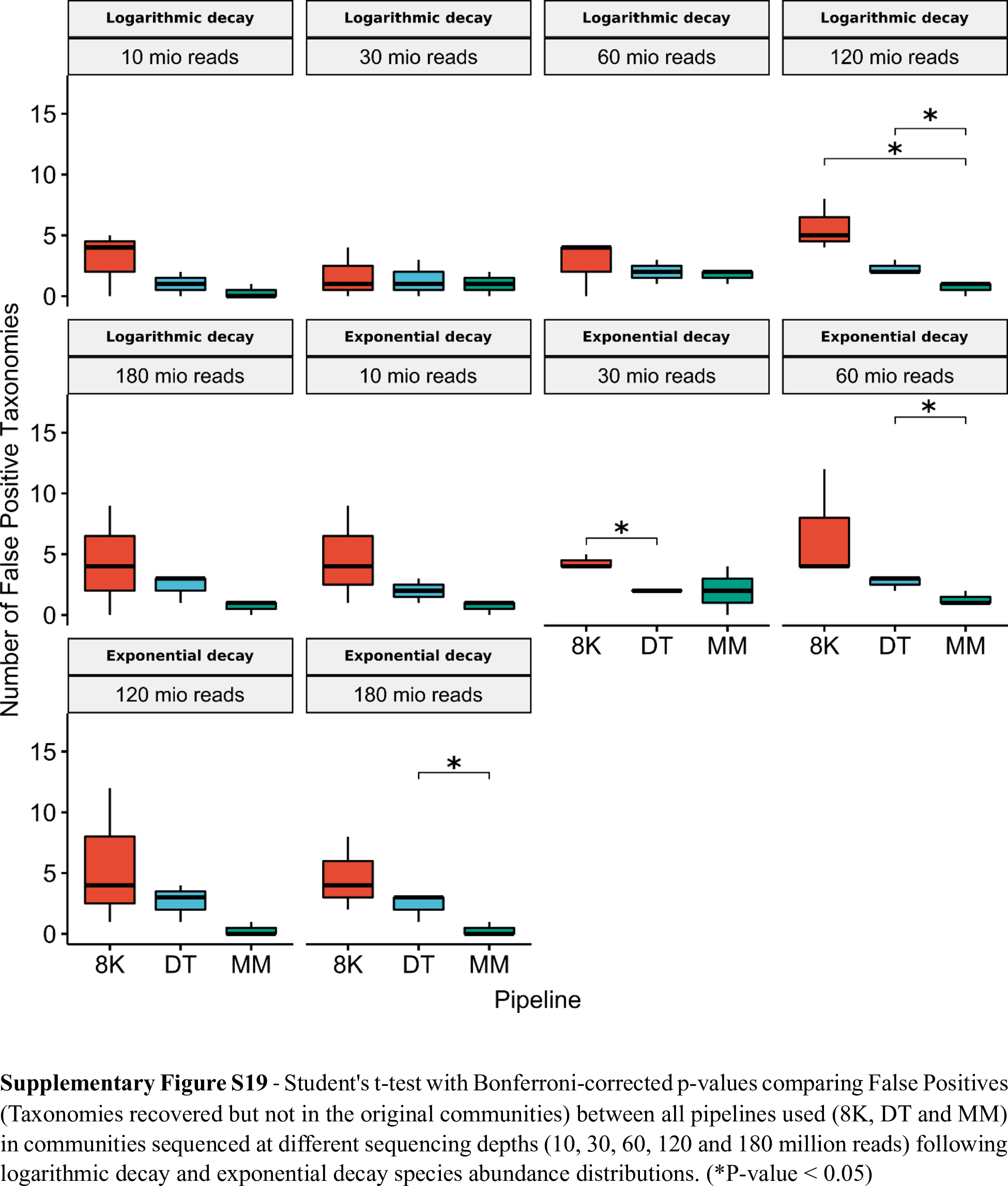

Supplement: S19 Fig — (*P-value < 0.05) (TIF) [file pcbi.1012530.s019.tif]

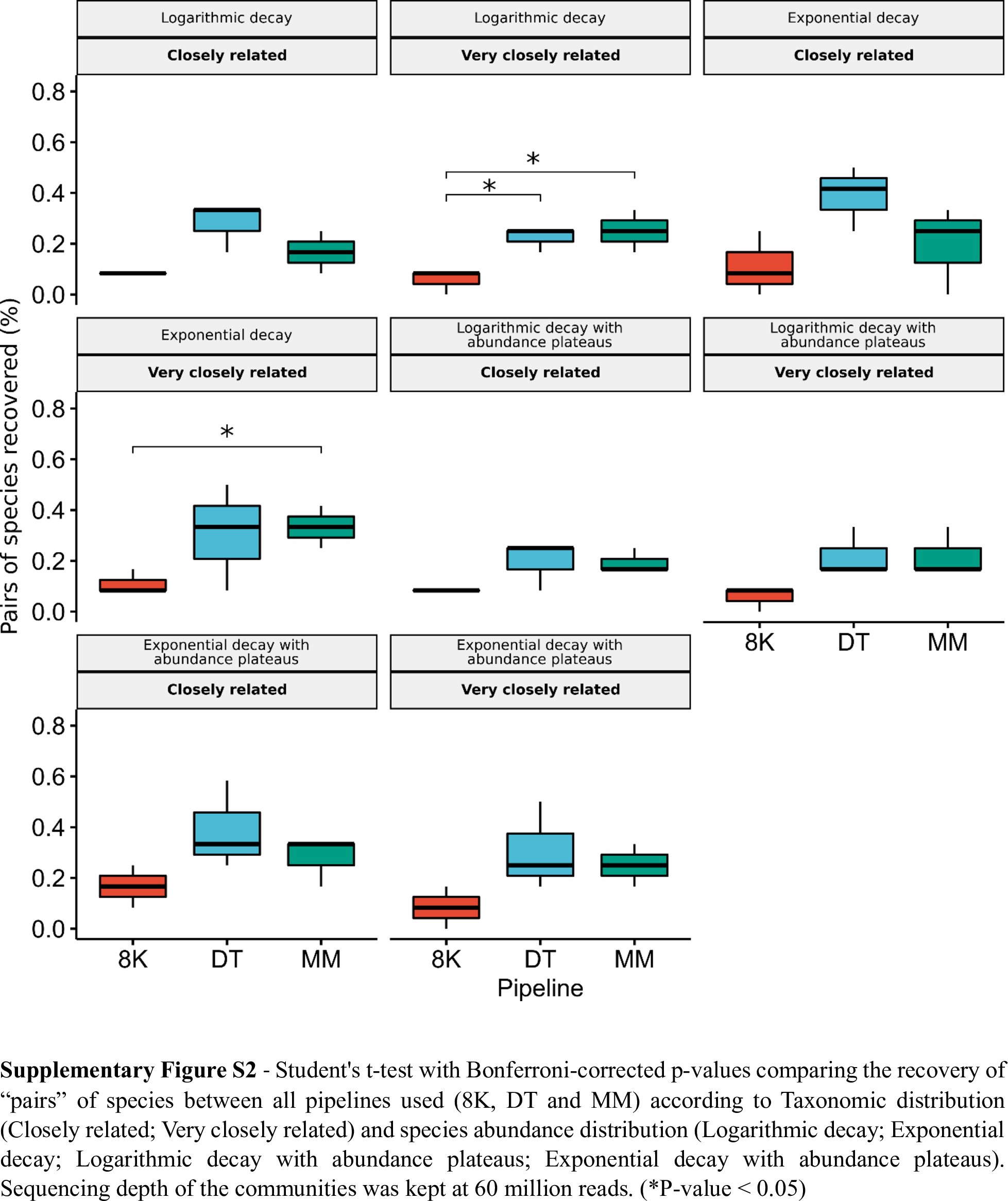

Supplement: S20 Fig — Sequencing depth of the communities was kept at 60 million reads. (*P-value < 0.05) (TIF) [file pcbi.1012530.s020.tif]

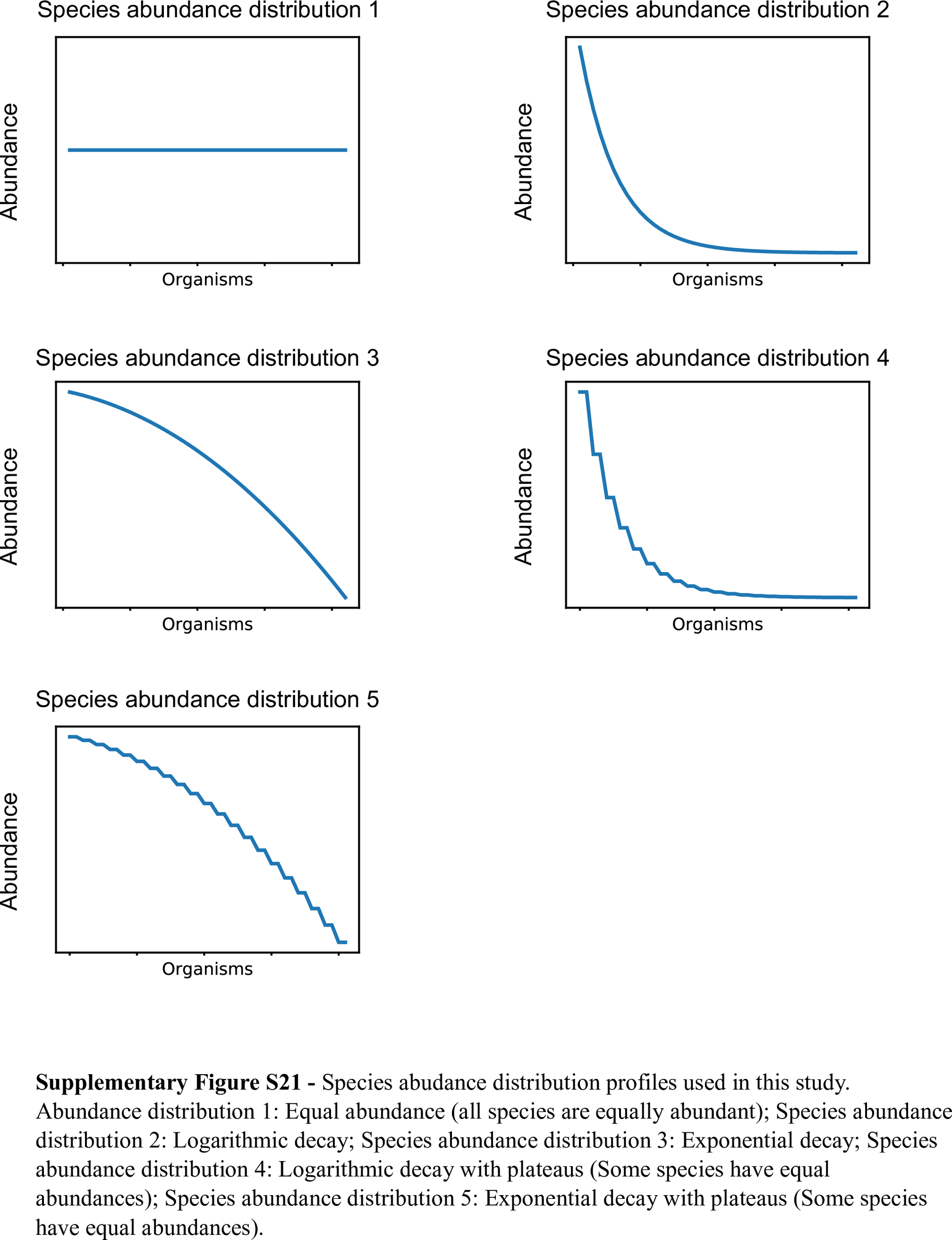

Supplement: S21 Fig — Abundance distribution 1: Equal abundance (all species are equally abundant); Species abundance distribution 2: Logarithmic decay; Species abundance distribution 3: Exponential decay; Species abundance distribution 4: Logarithmic decay with plateaus (Some species have equal abundances); Species abundance distribution 5: Exponential decay with plateaus (Some species have equal abundances). (TIF) [file pcbi.1012530.s021.tif]

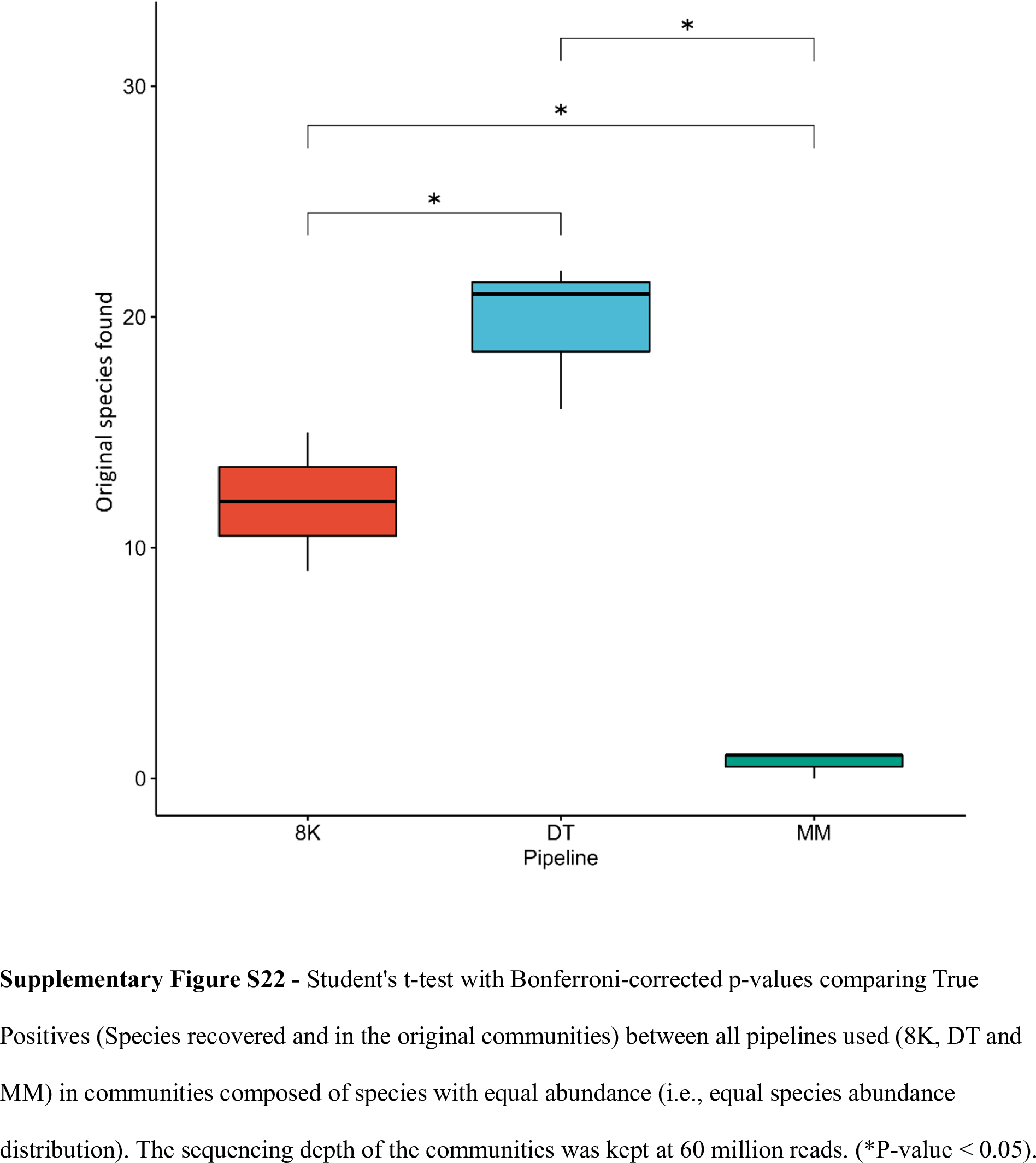

Supplement: S22 Fig — The sequencing depth of the communities was kept at 60 million reads. (*P-value < 0.05). (TIF) [file pcbi.1012530.s022.tif]
