# Supplementary material for: Simulation of 69 microbial communities indicates sequencing depth and false positives are major drivers of bias in prokaryotic metagenome-assembled genome recovery
Source: PLoS Comput Biol. 2024 Oct 22;20(10):e1012530. doi: 10.1371/journal.pcbi.1012530 (PMC11530072; doi:10.1371/journal.pcbi.1012530)
Supplement: Attachment — Submitted filename: PlosReview.pdf [file pcbi.1012530.s060.pdf]

Review of the Manuscript: "Simulation of 69 microbial communities indicates sequencing depth and false positives are major drivers of bias in Prokaryotic metagenome-assembled genome recovery".

September 12, 2023

Keywords Metagenomics · MAG assembly · Benchmark

Contents

1 Main Points of the review **1**

2 Almost section-by-section review **2**

2.1 Results Section 2

2.1.1 Genome Metrics 2

2.1.2 Assembly Results versus control variables 2

2.1.3 Overall Performance 3

2.1.4 Comments 3

2.2 Discussion Section 3

2.3 Methods Section 3

2.3.1 Sampling Depth 4

2.3.2 Taxonomic composition 4

2.3.3 Eveness 4

2.3.4 Comparison with literature 5

3 Conclusion **5**

1 Main Points of the review

I'll start by listing the strength and weakness points of the submitted article. In the next sections I will then go into the details of what is worth to discuss in details in a section by section way.

The strength of the article are mainly two. The first is that, the available benchmark dataset are not as heterogeneous as the samples that researchers daily deal with. Thus, designing a study where relevant variables such as species abundance, taxonomic relatedness and sequencing depth control how the synthetic communities are produced can be a valuable resource for the community. The second strength is the particular attention of the authors to include linear or multi-cromosomal genomes which are not so abundant in real microbial communities but, still, they may unavoidably

appear in an experimental sample. Even better, the authors also include fungal and eukariotic genomes to their mock samples making them even more realistic. These aspect are properly discussed in the discussion section. Overall, the research question explored in this study is of particular interest to the community of metagenomic binning tool developers and users and is worth to be investigated.

The weakness of the work mainly consist in the presentation of the results and the methods section, and comparison with available literature. In first place, the results section tends to be a long list of observation regarding the experiments, where many numbers are provided without a systematic organization and presentation. However, since there are coherent and incoherent patterns from the outcome of the study, this is not easy to achieve and I will discuss it in the appropriate section. In second place, we find the main weakness point of the work, which is the design of the microbial communities. The authors have designed communities controlling sequencing depth (they account for this in the right way and explore the right range) and vary what they refer to "eveness" (which is species abundance distribution) and taxonomic relatedness. I find that the employed definitions lack, in the case of species abundances, a knowledge of the main species abundance distribution in (microbial) ecology and that taxonomic relatedness relies on an obscure definition which, maybe due to my limits, i can not completely understand. Also, i would suggest to explore a wider ranges in the number of genomes to build a given mock community. Thus introducing a more quantitative approach may be also helpful to compare the dataset with former published benchmarks such as the CAMI dataset, to make crystal clear that the created dataset overcomes limitations of the former ones.

Minor limitations concern the organization of the manuscript. I find that aggregating some subsection and checking again for writing typos can be beneficial for the paper's quality.

While the paper provides valuable recommendations, it could benefit from more concrete implementation details. Offering step-by-step guidance or examples of how to apply the proposed methodologies would make it easier for readers to adopt these practices.

2 Almost section-by-section review

The last phrase that should recap the main findings is incomplete: *"Our data indicates the scientific community should their findings from MAG recovery, especially when asserting novel species or metabolic traits"*. The introduction is appropriate and I do not have major improvements to propose. I will directly go to reviewing the results.

2.1 Results Section

2.1.1 Genome Metrics

The *Genome Metric* section recapitulates some quantitative measures of the recovered genomes such as completeness. First, I would suggest to also report other relevant quantities in the field of comparative genomics, such as number of contigs, genome size, GC content, completeness, contamination. Each of this quantities can be easily measured with standard tools such as CheckM [8] and some python/bash scripting. It should be made clear if the reported quantities in the text are median or averages, to ease comparison with figures. Typically, boxplot employ median as centrality measure and, coherently the text should report the 50% IQR range. ON the other hand, if the boxplot employs the mean the text should report average and standard deviations.

Since also the recovery of 16S rRNA gene is a proxy for the goodness of the obtained genomes, I would include 16S rRNA metric in the genomic metrics section, adding to the table how many different 16S have been produced by each pipeline, how many of them could be classified at species/genus level and how many copies per genome they found. I would like to see some discussion about the pipeline performance for these metrics. Plus, the authors report (line 115,116) that the pielines assembled 16s rRNA sequences from archaeal and eukariotic genomes, opposed to what's reported in the discussion (lines 329,330,331). They should clarify this point.

2.1.2 Assembly Results versus control variables

To avoid the reader to continuously go back and forth from one section to another, I would organize the section according to the scientific question the work address.

The first subsection section of the results should be focused, respectively on the effect of the control variable on the assembly performance: sequencing depth, taxonomic relatedness and species abundance. for example, I would aggregate section 2.3 and 2.7 and making them the 2.1. Second, I would dedicate a subsection to the effect of taxonomic relatedness on the MAG recovery, and comment how this variable affects the taxonomic resolution of the recover process. Third, I would write a section solely focusing on the effects of species abundance. In particular i would

focus on distinguishing which variables have -o not- , among the different pipelines, a coherent effect on the MAG recovery, distill practical advice for the metagenomics community. Especially in the reorganization of results I strongly recommend a more succinct and explanatory language and organization of the text, due to the vast amount of the produced results. The section about taxonomic relatedness should be implemented by handling the results reported in section 2.8 of the main text and results from other sections . Since the authors claim that they have very mild effect from species abundance and a strong effect on MAG retrieval with sequencing depth (also with contrasting patterns), i would suggest to avoid so many barplot and, instead to have a single parplot where the most relevant variable (sampling depth) is used for the x axis and on the y axis one has the number of recovered MAGs, possibly normalized by the number of MAGs employed to build the mock community.

2.1.3 Overall Performance

One subsection should be dedicated to the effect of the control variables on the True/False positive effects, which in this context are appropriate metrics. Overall, other reported quantities should be organized in tabular form to ease the reader going through the results. Focusing on the presentation of this section (see figures 5) i would add to the barplot the number of MAGs actually employed to create the mock communities. Another way it may be to normalize the y-axis to this ground truth (which along the text is sometimes neglected) so that it represents percentage which are easier when thinking about comparing the results with the objective. Since especially in this section there is a lot of results to take in account, i would recommend to organize them in a tabular form, or including a heatmap where from which the dependence of TP/FP performance from the control variable is clear. In general i strongly recommend to refer to other papers published by the CAMI community see [7]

2.1.4 Comments

In a final subsection, I would allocate all the observations relative to the inclusion of non prokaryotic-DNA and to peculiar form of genomic structure such as linear genomes or multi-chromosomal genomes. This issues deserve a their own section, since they may be strongly appreciated by the community since the available standard benchmarking datasets do not take in account for this relevant points. I would also consider to move section 2.7, as to include it in the "species detection" section, or to allocate these results in the results section concerning sequencing depth effect on overall performance. Also, I have an issue in the definition of the mean relative abundance in a given community. Since abundances are constrained to sum to one, given $v_{i=1,...,G}$ the G relative abundances, we have for a given community

$$\overline{v} = \frac{1}{G}\sum_{i=1}^{G} v_i = \frac{1}{G} \tag{1}$$

When averaging this quantity among different communities we just get the harmonic mean of the number of species, which is not a proxy for abundance but, instead, of community richness. Thus, I would avoid to refer to this quantity when discussing about rare species. To compare relative abundances of a rare species v_{rare} I would compare that to the relative abundance of the most abundant species v_{top} by their ratio $\nu_{rare} = v_{rare}/v_{top}$, which makes sense [3]. Also, i would check how coverage is a proxy for relative abundance.

2.2 Discussion Section

The first part of the discussion section (lines 326-245) appropriately address the points of the manuscript. The only point, is that authors claim that they do not have eukaryotic genomic content contamination while, as mentioned in the genomes metric 2.1.1, they report that the assembly process lead to the classification of a significant number of 16S rRNA genes sequences. This seems to be a contradiction, which must be soled. I have comments about some cailms by the authors regarding the existing literature, at lines 385 and line 388. In the first case, authors report that bioreactors communities follow a log-decay (whatever it means, since there is no formula for that species abundance distribution) and in the second case they report for pristine-like communities exponential decay distribution. First, they do not provide any literature to support that affirmation. Second, the ecology literature report way more heterogeneous species abundance distributions which follow log-series, log-normal or power law like behaviour. But in the following section I will discuss better these points.

2.3 Methods Section

The bioinformatic workflow section dedicated to quality check, assembly and taxonomic classification the authors employed is overall appropriate to address their scientific question (figure 6, main text). However the first part of the

workflow, which is about the design of the microbial communities, is sometimes not clear and, in my opinion, fails to explore the relevant set of relevant species abundance distribution in microbial ecology. Plus, the number of genomes employed to simulate the mock communities is kept constant, while I think that, in a benchmark dataset this needs to be considered as a relevant control variable Generally, the quantitative framework employed by the authors is poor, and need to be heavily improved.

2.3.1 Sampling Depth

The definition employed by the authors of the sampling depth is appropriate.

2.3.2 Taxonomic composition

The criteria by which they define taxonomic relatedness remain somewhat ambiguous to me. When attempting to grasp the underlying rationale behind their procedure for generating a community, for instance, one comprising 45 MAGs starting from the initial set of 126 genomes, I encountered difficulties in comprehending the methodology. To facilitate a deeper understanding, it would be highly beneficial if the authors could provide a practical example, potentially in the supplementary figures (which I may not have received), complete with a graphical representation to elucidate the procedure's reasoning. Presently, the determination of 'very closely related' relies on species belonging to the same genus, corresponding to a taxonomic distance of 2 on the taxonomic tree. However, since taxonomic distance can be measured in several ways, including using a network approach where the distance between two species of the same genus is 2, or through methods like average nucleotide identity (which is also relevant for MAGs reconstruction), it would be valuable for the authors to clarify the specific metric employed. In the end, I do not understood what the author mean by "closely related" species but I was not able to understand what does it means at the community level: this makes the reproducibility of the results really hard. Additionally, introducing a quantitative measure of taxonomic relatedness within a community, such as computing the average taxonomic distance or average nucleotide identity among species in the community, could enhance the clarity of their taxonomic criteria. In general, since several genomic similarity measures are available (average nucleotide identity ANI [10], 16S rRNA similarity [5]) it is demanding to improve the taxonomic relatedness keeping in mind that there exists a wealth of literature about phylogenetic diversity in ecological communities [2], [1]. Per se, the proposed criteria are not wrong, but should be integrated with a more careful phylogenetic analysis. In the end, the authors should also explore a wider range for the number of genomes in the community.

2.3.3 Eveness

The authors expose the quantities involved in the article in a basic way. They do not make a clear distinction between species abundance and "eveness" simply confounding them. What they mean by "eveness" is what is known in quantitative ecology as species abundance distribution. From such quantity one can introduce some heterogeneity measure (such as Hill numbers [2]) to asses the eveness of the underlying SAD. Simply, there is no analytical formula for the species distributions they used to sample relative abundances. More, I would avoid to employ the plateaus-like distribution the authors proposed, since they do not appear in the ecological literature, and introduce another level of complexity to the results. Even if I obviously know what an exponential distribution is from the text I can not say what the authors mean by "logarithmic decay". Is it a log-normal distribution a log-series distribution or what else? From my perspective there is no real reason to choose the exponential distribution, see the discussion in 2.2.

Thus I will need to see some mathematical details, otherwise the methodology will remain obscure. I will sketch some quantities that background suggests me that the authors should consider in order to improve the quantitative side of their study design. In theoretical ecology, eveness and species abundance are two different but related quantities: it's crucial to distinguish evenness from species abundance and provide a clear methodology for its evaluation. Researchers can select a parametric distribution, such log-normal, or power law, which are commonly used in ecological literature [4], [6], [9]. These distributions can serve as the basis for simulating communities with varying evenness levels. To assess evenness of the species abundance distribution one can set different parameters of the target distribution, sample abundances and normalize them. From here one can calcualte the relative abundance and the entropy (eveness of the abundance profile). Now, we just employ the most known Hill number, the Shannon entropy, which reads:

$$H_{\textit{Rel. Abundance}} = -\sum_{g=1}^{G} p_g \log p_g \tag{2}$$

With which one can implement in the following diversity index:

$$D_{\textit{Rel. Abundance}} = e^{H_{\textit{Rel. Abundance}}} \tag{3}$$

For example, in the case of uniform distribution one finds that this is the number of genomes G. Applying this quantitative framework would lead to the redesign of the mock communities sampling and to e new run of all the bioinformatic workflow.

2.3.4 Comparison with literature

Given the authors' assertion that they are exploring different aspects compared to established benchmark datasets like CAMI, it would be insightful to investigate the distinctions between their generated communities and those in CAMI. This comparative analysis can provide valuable context and help illustrate the uniqueness of their approach. Therefore, it could be beneficial for the authors to consider applying the definitions of evenness and taxonomic relatedness to CAMI datasets. By doing so, they could potentially reveal quantitative differences in evenness or taxonomic relatedness, shedding light on the specific characteristics that differentiate their communities. Such an analysis could enhance our understanding of the performance and relevance of their generated communities within the field of metagenomics.

References

[1] Benjamin Allen, Mark Kon, and Yaneer Bar-Yam. "A new phylogenetic diversity measure generalizing the Shannon index and its application to phyllostomid bats". In: *The American Naturalist* 174.2 (2009), pp. 236–243.

[2] Anne Chao, Chun-Huo Chiu, and Lou Jost. "Phylogenetic diversity measures based on Hill numbers". In: *Philosophical Transactions of the Royal Society B: Biological Sciences* 365.1558 (2010), pp. 3599–3609.

[3] Juan José Egozcue and Vera Pawlowsky-Glahn. "Compositional data: the sample space and its structure". In: *Test* 28.3 (2019), pp. 599–638.

[4] Jacopo Grilli. "Macroecological laws describe variation and diversity in microbial communities". In: *Nature communications* 11.1 (2020), p. 4743.

[5] Minseok Kim, Mark Morrison, and Zhongtang Yu. "Evaluation of different partial 16S rRNA gene sequence regions for phylogenetic analysis of microbiomes". In: *Journal of microbiological methods* 84.1 (2011), pp. 81–87.

[6] Robert Marsland III, Wenping Cui, and Pankaj Mehta. "A minimal model for microbial biodiversity can reproduce experimentally observed ecological patterns". In: *Scientific reports* 10.1 (2020), p. 3308.

[7] Fernando Meyer et al. "Tutorial: assessing metagenomics software with the CAMI benchmarking toolkit". In: *Nature protocols* 16.4 (2021), pp. 1785–1801.

[8] Donovan H Parks et al. "CheckM: assessing the quality of microbial genomes recovered from isolates, single cells, and metagenomes". In: *Genome research* 25.7 (2015), pp. 1043–1055.

[9] Igor Volkov et al. "Patterns of relative species abundance in rainforests and coral reefs". In: *Nature* 450.7166 (2007), pp. 45–49.

[10] Seok-Hwan Yoon et al. "A large-scale evaluation of algorithms to calculate average nucleotide identity". In: *Antonie Van Leeuwenhoek* 110 (2017), pp. 1281–1286.
