# Supplementary material for: Simulation of 69 microbial communities indicates sequencing depth and false positives are major drivers of bias in prokaryotic metagenome-assembled genome recovery
Source: PLoS Comput Biol. 2024 Oct 22;20(10):e1012530. doi: 10.1371/journal.pcbi.1012530 (PMC11530072; doi:10.1371/journal.pcbi.1012530)
Supplement: Attachment — Submitted filename: 01_PointbyPointReplytoReviewers_v6.pdf [file pcbi.1012530.s061.pdf]

Dear Editor,

We appreciate the comments and points raised by the Editor.
We believe all comments have been considered/discussed in the revised version.

Briefly, we assigned sequential numbers to each reviewer's comments. We replied to each comment (the number after each 'Reply' corresponds to the comment it refers to).
We highlighted the altered text in the manuscript using the track changes option to facilitate the reviewers' and your work.
Additionally, we updated all tables, figures, and supplementary tables to reflect the change from "evenness" to "species abundance".
We hope the point-by-point replies are clear and address all the Editor's comments, and we are looking forward to receiving the reply.

Sincerely,
Ulisses Nunes da Rocha

Editor

Reviewer 1
Comment 1
The last phrase that should recap the main findings is incomplete: "Our data indicates the scientific community should their findings from MAG recovery, especially when asserting novel species or metabolic traits". The introduction is appropriate, and I do not have major improvements to propose. I will directly go to reviewing the results.

Reply 1: We thank the reviewer for their comment. We have added the word "curate" to the sentence in line 37.

Comment 2
The Genome Metric section recapitulates some quantitative measures of the recovered genomes such as completeness. First, I would suggest to also report other relevant quantities in the field of comparative genomics, such as number of contigs, genome size, GC content, completeness, contamination.

Reply 2: We thank the reviewer for their suggestion. We have now included these parameters in the Supplementary Table S01.

Comment 3
t should be made clear if the reported quantities in the text are median or averages, to ease comparison with figures. Typically, boxplot employ median as centrality measure and, coherently the text should report the 50% IQR range. ON the other hand, if the boxplot employs the mean the text should report average and standard deviations.

Reply 3: We thank the reviewer for their comment. In our study, we use the means. The averages and standard deviations have been included throughout the newly formatted results section.

Comment 4

Since also the recovery of 16S rRNA gene is a proxy for the goodness of the obtained genomes, I would include 16S rRNA metric in the genomic metrics section, adding to the table how many different 16S have been produced by each pipeline, how many of them could be classified at species/genus level and how many copies per genome they found. I would like to see some discussion about the pipeline performance for these metrics. Plus, the authors report (line 115,116) that the pielines assembled 16s rRNA sequences from archaeal and eukariotic genomes, opposed to what's reported in the discussion (lines 329,330,331). They should clarify this point.

Reply 4: We thank the reviewer for their comment. The requested 16S rRNA metrics have been added to Supplementary Table S03. Additionally, the total and unique number of 16S rRNA sequences has been included in the main manuscript in lines 146-151. The recovery of eukaryotic rRNA sequences has also been addressed in lines 262-263 and 319-322 of the results section. Briefly, the reconstruction of 16S rRNA sequences was done independently of the MAG recovery. Lines 262-263 also should make clear to the reader that while we do recover 16S rRNA sequences from the simulated libraries using a tool specifically designed for that purpose, we do not observe any effect of the inclusion of eukaryotic genomes during the reconstruction of bacterial and archaeal MAGs.

Comment 5

The first subsection section of the results should be focused, respectively on the effect of the control variable on the assembly performance: sequencing depth, taxonomic relatedness and species abundance. for example, I would aggregate section 2.3 and 2.7 and making them the 2.1

Reply 5: We thank the reviewer for their comment. Following the reviewer's suggestions, we have completely restructured the results section. Additionally, we have included summarized experimental explanations throughout the results section (e.g., lines 120-137) that should improve the manuscript's readability and clarity. The new results format starts with the general description of the MAGs recovery results, followed by the sections: "Impact of Sequencing Depth on the Number of MAG Recovery", "Influence of Taxonomic Relatedness on MAG Recovery", "Effects of Species Abundance Profile on MAG Recovery", "Analysis of True and False Positive Effects", "Detection Limit", "Special Genome Structures and Recovery Challenges". We have also added text in lines 423-439 of the manuscript to emphasize the main takeaways of the study.

Comment 6

Second, I would dedicate a subsection to the effect of taxonomic relatedness on the MAG recovery, and comment how this variable affects the taxonomic resolution of the recover process.

Reply 6: We thank the reviewer for their comment. Following the reviewer's suggestions, we have completely restructured the results section. We have also added text in lines 165-185 and 434-439 of the manuscript to emphasize the effect of taxonomic relatedness on MAG recovery.

Comment 7

Third, I would write a section solely focusing on the effects of species abundance. In particular I would focus on distinguishing which variables have -o not- , among the different pipelines, a coherent effect on the MAG recovery, distill practical advice for the metagenomics community. Especially in the reorganization of results I strongly recommend a more succinct and explanatory language and organization of the text, due to the vast amount of the produced results. The section about taxonomic relatedness should be implemented by handling the results reported in section 2.8 of the main text and results from other sections. Since the authors claim that they have very mild effect from species abundance and a strong effect on MAG retrieval with sequencing depth (also with contrasting patterns), I would suggest to avoid so many barplot and, instead to have a single parplot where the most relevant variable (sampling depth) is used for the x axis and on the y axis one has the number of recovered MAGs, possibly normalized by the number of MAGs employed to build the mock community.

Reply 7: We thank the reviewer for their comment. We agree with the reviewer that presenting such an amount of results is challenging. As mentioned in previous replies, we have completely restructured the results section. Additionally, we have included summarized experimental explanations throughout the results section (e.g., lines 120-137) that should improve the manuscript's readability and clarity. The text has been added in lines 432-439 of the manuscript, emphasizing practical advice for the metagenomics community. Finally, we have also included two new figures (Figure 1 and Figure 2) that we believe more clearly summarize the main results observed.

Comment 8

One subsection should be dedicated to the effect of the control variables on the True/False positive effects, which in this context are appropriate metrics. Overall, other reported quantities should be organized in tabular form to ease the reader going through the results. Focusing on the presentation of this section (see figures 5) i would add to the barplot the number of MAGs actually employed to create the mock communities. Another way it may be to normalize the y-axis to this ground truth (which along the text is sometimes neglected) so that it represents percentage which are easier when thinking about comparing the results with the objective. Since especially in this section there is a lot of results to take in account, i would recommend to organize them in a tabular form, or including a heatmap where from which the dependence of TP/FP performance from the control variable is clear. In general i strongly recommend to refer to other papers published by the CAMI community see [7]

Reply 8: We thank the reviewer for the comments and consideration of our manuscript. Following the reviewer's suggestion, we created a dedicated results section to describe the effects on the True and False positives recovery (lines 208-213). Additionally, we have created Figure 1b,c,e,f to summarize better the effects of the community profiles on the TP and FP recovery. However, it is worth mentioning that our study focuses on specific and punctual variables involving the recovery of metagenome-assembled genomes (MAGs). Specifically, we focus on the sequencing depth, taxonomic relatedness, and abundance distribution of microbiome community members and dissect these variables' individual and nuanced influences on MAGs. Unlike the CAMI benchmarks, which broadly assess and focus on methods and tools for assembly, taxonomic profiling, and binning in metagenomics, our work aims to provide a fine-resolution understanding of the mentioned specific influences. Although we test the parameters on different recovery pipelines, it only supports the findings for the influences of sequencing depth, taxonomic relatedness, and abundance distribution. Consequently, we also used a boxplot data visualization (Figure 4) to focus on the isolated impact of each variable on TP recovery. Boxplots can effectively highlight differences between groups and allow us to draw more precise indications of the variable under investigation. We have made the results available in tabular format in the Supplementary Table S2. We also included a brief explanation of the defined groups before the results in lines 120-137 to guide the reader through the results more efficiently.

Comment 9

In a final subsection, I would allocate all the observations relative to the inclusion of non prokaryotic-DNA and to peculiar form of genomic structure such as linear genomes or multi-chromosomal genomes. This issues deserve a their own section, since they may be strongly appreciated by the community since the available standard benchmarking datasets do not take in account for this relevant points. I would also consider to move section 2.7, as to include it in the "species detection" section, or to allocate these results in the results section concerning sequencing depth effect on overall performance.

Also, I have an issue in the definition of the mean relative abundance in a given community. Since abundances are constrained to sum to one, given vi=1,...,G the G relative abundances, we have for a given community. When averaging this quantity among different communities we just get the harmonic mean of the number of species, which is not a proxy for abundance but, instead, of community richness. Thus, I would avoid to refer to this quantity when discussing about rare species. To compare relative abundances of a rare species vrare I would compare that to the relative abundance of the most abundant species vtop by their ratio vrare = vrare/vtop, which makes sense [3]. Also, I would check how coverage is a proxy for relative abundance.

Reply 9: We thank the reviewer for their comment. Following the reviewer's suggestion, we have now completely restructured the presentation of the Results section so its readability is improved. The results of linear and multi-chromosomal genomes now have their subsection in the manuscript (Special Genome Structures and Recovery Challenges) (Lines 297-313). Additionally, we detailed the equation used to construct species abundance distribution profiles in the methods section (Line

471). We have also added text in Lines 378-384 of the discussion section to improve further clarity in the definitions for species abundance with concrete examples.

Comment 10

The first part of the discussion section (lines 326-245) appropriately address the points of the manuscript. The only point, is that authors claim that they do not have eukaryotic genomic content contamination while, as mentioned in the genomes metric 2.1.1, they report that the assembly process lead to the classification of a significant number of 16S rRNA genes sequences. This seems to be a contradiction, which must be soled. I have comments about some claims by the authors regarding the existing literature, at lines 385 and line 388. In the first case, authors report that bioreactors communities follow a log-decay (whatever it means, since there is no formula for that species abundance distribution) and in the second case they report for pristine-like communities exponential decay distribution. First, they do not provide any literature to support that affirmation. Second, the ecology literature report way more heterogeneous species abundance distributions which follow log-series, log-normal or power law like behaviour. But in the following section I will discuss better these points.

Reply 10: We thank the reviewer for their comment. The reconstruction of 16S rRNA sequences was done by the ssu_finder function of CheckM, which was done independently of the MAG recovery. We have modified the text in lines 319-322 to make it clear to the reader that while we do recover 16S rRNA sequences from the simulated libraries using a tool specifically designed for that purpose, we do not observe any effect of the inclusion of eukaryotic genomes during the reconstruction of bacterial and archaeal MAGs. We have also added the tool used to recover 16S rRNA sequences in the methods section in Lines 563-564 of the manuscript.

Furthermore, we have added text in lines 378-384 to improve clarity in the definition of the different species abundance distribution profiles and how they relate to some environments. Briefly, logarithmic decay species abundance distribution reflects communities where a few species are very dominant and in higher numbers with a massive drop in species abundance of the remaining members of the community. This is frequently observed in bioreactors containing enriched cultures. In contrast, exponential decay species abundance distribution represents communities where several species are in higher numbers, and only a few have very low abundance. This is usually the case in pristine natural environments. The inclusion of abundance plateaus was done to determine MAG recovery when species had exactly the same abundance at different abundance levels.

Comment 11

The bioinformatic workflow section dedicated to quality check, assembly and taxonomic classification the authors employed is overall appropriate to address their scientific question (figure 6, main text). However the first part of the workflow, which is about the design of the microbial communities, is sometimes not clear and, in my opinion, fails to explore the relevant set

of relevant species abundance distribution in microbial ecology. Plus, the number of genomes employed to simulate the mock communities is kept constant, while I think that, in a benchmark dataset this needs to be considered as a relevant control variable Generally, the quantitative framework employed by the authors is poor, and need to be heavily improved.

Reply 11: We thank the reviewer for their comment. We have improved the workflow figure (Figure 5, now) and added a detailed rationale linked to microbial ecology concepts. A constant number of species' abundance was used to compare the limits of detection and indicate which species' abundance distribution would affect MAG recovery. Studies have shown that we can recover roughly 10 MAGs per metagenomic library on average (Bandla et al., 2020). Thus, we constructed communities with almost four times more species than what we usually recover. We agree that varying the number of species is relevant in the field of ecology but falls outside the scope of this work. Nevertheless, we have added text in lines 423-425, highlighting this precise point and mentioning that future studies using simulated microbial communities may address the issues of species richness.

Comment 12

The criteria by which they define taxonomic relatedness remain somewhat ambiguous to me. When attempting to grasp the underlying rationale behind their procedure for generating a community, for instance, one comprising 45 MAGs starting from the initial set of 126 genomes, I encountered difficulties in comprehending the methodology. To facilitate a deeper understanding, it would be highly beneficial if the authors could provide a practical example, potentially in the supplementary figures (which I may not have received), complete with a graphical representation to elucidate the procedure's reasoning. Presently, the determination of' very closely related' relies on species belonging to the same genus, corresponding to a taxonomic distance of 2 on the taxonomic tree. However, since taxonomic distance can be measured in several ways, including using a network approach where the distance between two species of the same genus is 2, or through methods like average nucleotide identity (which is also relevant for MAGs reconstruction), it would be valuable for the authors to clarify the specific metric employed. In the end, I do not understood what the author mean by "closely related" species but I was not able to understand what does it means at the community level: this makes the reproducibility of the results really hard. Additionally, introducing a quantitative measure of taxonomic relatedness within a community, such as computing the average taxonomic distance or average nucleotide identity among species in the community, could enhance the clarity of their taxonomic criteria. In general, since several genomic similarity measures are available (average nucleotide identity ANI [10], 16S rRNA similarity [5]) it is demanding to improve the taxonomic relatedness keeping in mind that there exists a wealth of literature about phylogenetic diversity in ecological communities [2], [1]. Per se, the proposed criteria are not wrong, but should be integrated with a more careful phylogenetic analysis. In the end, the authors should also explore a wider range for the number of genomes in the community.

Reply 12: We thank the reviewer for their comment. We agree with the reviewer that multiple methods of defining taxonomic relatedness exist. We have added a more detailed explanation on the selection of species based on taxonomic relatedness in Lines 457-467 of the main manuscript. The number of species per community was determined to address the issue of lack of true biological replicates when studying microbial communities. It is also worth mentioning that Supplemental_Table_S30 has the specific species used as Closely related and Very Closely Related for the different generated communities.

In regards to the rationale behind taxonomic distribution, we did not use phylogenetic distance but rather simple taxonomic classification. Although at 0.95 ANI distance, we would be able to differentiate species, to the best of our knowledge, this was not rigorously studied across different taxonomic levels. Therefore, choosing species based on fixed quantitative measurements would not indicate if the distances reflect different taxonomic levels. Nevertheless, the use of ANI distances is indeed interesting and should be applied in future studies.

The study of phylogenetic diversity in ecological communities is important but falls outside the scope of this study.

Comment 13

The authors expose the quantities involved in the article in a basic way. They do not make a clear distinction between species abundance and "eveness" simply confounding them. What they mean by "eveness" is what is known in quantitative ecology as species abundance distribution. From such quantity one can introduce some heterogeneity measure (such as Hill numbers [2]) to asses the eveness of the underlying SAD. Simply, there is no analytical formula for the species distributions they used to sample relative abundances. More, I would avoid to employ the plateaus-like distribution the authors proposed, since they do not appear in the ecological literature, and introduce another level of complexity to the results. Even if I obviously know what an exponential distribution is from the text I can not say what the authors mean by "logarithmic decay". Is it a log-normal distribution a log-series distribution or what else? From my perspective there is no real reason to choose the exponential distribution, see the discussion in 2.2. Thus I will need to see some mathematical details, otherwise the methodology will remain obscure. I will sketch some quantities that background suggests me that the authors should consider in order to improve the quantitative side of their study design. In theoretical ecology, eveness and species abundance are two different but related quantities: it's crucial to distinguish evenness from species abundance and provide a clear methodology for its evaluation. Researchers can select a parametric distribution, such log-normal, or power law, which are commonly used in ecological literature [4], [6], [9]. These distributions can serve as the basis for simulating communities with varying evenness levels. To assess evenness of the species abundance distribution one can set different parameters of the target distribution, sample abundances and normalize them. From here one can calcualte the relative abundance and the entropy (eveness of the abundance profile). Now, we just employ the most known Hill number, the Shannon entropy (…).

Reply 13: We thank the reviewer for their comment. To prevent confusion, we have changed evenness to species abundance throughout the entire text and supplementary materials. Regarding

the issue of species abundance distribution, we have added text in lines 122-134, 378-384 to improve clarity in the definition of the different species abundance distribution profiles and how they relate to some environments. Moreover, we included additional details on the generation of the species abundance profiles in the methods section lines 489-502. Finally, the reader should find the exact abundances used for each species for each generated community profile in Supplemental Table S37. Supplemental Figure S21 also has a visual representation of the used rationale.

However, it is worth mentioning the rationale behind it. Briefly, logarithmic decay species abundance distribution reflects communities where a few species are very dominant and in higher numbers with a massive drop in species abundance of the remaining members of the community. This is frequently observed in bioreactors containing enriched cultures. In contrast, exponential decay species abundance distribution represents communities where several species are in higher numbers, and only a few have very low abundance. This is usually the case in pristine natural environments. The inclusion of abundance plateaus was done to determine MAG recovery when species had exactly the same abundance at different abundance levels.

Regarding the comment, "Researchers can select a parametric distribution, such log-normal, or power law, which are commonly (…)", we agree with the reviewer, but this falls outside the scope of this study since we are not simulating natural communities. Our intention is merely to educate the microbial ecology community that species abundance distribution influences MAG recovery.

Here, we define the relative abundance of a species as the number of mapped reads in the MAG divided by the total number of reads in the sample.

Comment 14

Given the authors' assertion that they are exploring different aspects compared to established benchmark datasets like CAMI, it would be insightful to investigate the distinctions between their generated communities and those in CAMI. This comparative analysis can provide valuable context and help illustrate the uniqueness of their approach. Therefore, it could be beneficial for the authors to consider applying the definitions of evenness and taxonomic relatedness to CAMI datasets. By doing so, they could potentially reveal quantitative differences in evenness or taxonomic relatedness, shedding light on the specific characteristics that differentiate their communities. Such an analysis could enhance our understanding of the performance and relevance of their generated communities within the field of metagenomics.

Reply 14: we thank the reviewer for their comment. While it is interesting to assess the quantitative differences in species abundances and taxonomic relatedness, this task falls outside the scope of this study.

Reviewer 2

Comment 1

The generation of Metagenome-Assembled Genomes (MAGs) from individual microbes within complex samples is a critical step for identifying the community members present and their functional capacity within a metagenomics sample. Previous work has shown that intrinsic parameters of the sample or the sequencing can bias MAG generation, which would significantly alter the interpretation of a metagenomics experiment's results. The manuscript by da Rocha et al aims to investigate bias in tools used for MAG generation. The authors generated in silico microbial communities, allowing them to test three MAG generation pipelines for the effect of sample evenness, sequencing depth, and taxonomic relatedness on MAG recovery. This work is of significant interest to individuals working with metagenomics data, as it tests the influence of these factors on bias in MAG recovery as well as tests the accuracy of several MAG generation pipelines on simulated data.

Reply 1: We thank the reviewer for their comment and appreciate their positive feedback.

Comment 2

Overall, the authors found that the DAS Tool (DT) pipeline had the highest accuracy of MAG generation pipelines tested. The authors also found other associations between the number of MAGs recovered and other parameters, although there were few consistent trends. Of note, the statistical approach used is not suited for the number of comparisons made in this analysis, making it difficult to interpret these results. In addition, the language of the manuscript could be revised to ease interpretation of the results.

Reply 2: Following the reviewer's suggestions, we have completely restructured the results section. Additionally, we have included summarized experimental explanations throughout the results section (e.g., lines 120-137) that should improve the manuscript's readability and clarity. The new results format starts with the general description of the MAGs recovery results, followed by the sections: "Impact of Sequencing Depth on the Number of MAG Recovery", "Influence of Taxonomic Relatedness on MAG Recovery", "Effects of Species Abundance Profile on MAG Recovery", "Analysis of True and False Positive Effects", "Detection Limit", "Special Genome Structures and Recovery Challenges". We have also added text in lines 423-439 of the manuscript to emphasize the main takeaways of the study. In our study, we compare results (Number of bins, True positives, and False positives) for each variable (sequencing depth, taxonomic relatedness, and species abundance). Additionally, we have included two new figures (Figure 1 and Figure 2) that should help guide the reader to interpret the results.

Comment 3

Throughout the paper, the authors use a Student's t test to test for significant differences between groups. However, a Student's t test is only valid for comparisons between two groups, and most of these comparisons involve three or more comparisons. Therefore, the statistics should be revised for this study to use an alternative test, such as a One-way ANOVA, instead.

Reply 3: We thank the reviewer for their comment. In our study, we compare results (Number of mags, True positives, and False positives) for each variable (sequencing depth, taxonomic relatedness, and species abundance). Consequently, we applied a pairwise t-test to test the effect of an isolated variable between two groups only, and thus, the t-test holds its statistical significance. For instance, when we compare the pipeline effect on the number of MAGs recovered, we set a fixed sequencing depth (e.g., 60 mio) and a fixed species abundance distribution (e.g., exponential decay), and perform the t-test pairwise on the pipelines, i.e., 8K vs. DT, 8K vs. MM, and DT vss MM.

Comment 4

While the authors draw many conclusions on the associations between sample evenness, sequencing depth, and taxonomic similarity, most of this data is presented in supplementary figures, with only one figure (Fig. 3) showing significant differences across these parameters. I think some of this data should be presented within the body of the manuscript as main figures to ease interpretation of the work.

Reply 4: We thank the reviewer for their comment. Figures were included in the main text based on the main takeaways of the study.

Comment 5

The text should be revised to more clearly explain the main takeaways of the paper. For example, more detailed explanation of the evenness models investigated should be included within the results section.

Reply 5: We thank the reviewer for their comment. We have restructured the results section to improve the manuscript's clarity. Additionally, we have added text in the main manuscript (Lines 378-384) to improve clarity about the rationale used to define the different species abundance distributions. You may find a detailed definition of species abundance in the methods section, lines 489-502. We included text in Lines 423-439 of the manuscript highlighting the main takeaways of the study.

Minor Comments

1. On line 35, the final sentence of the abstract is incomplete and should be revised.

Reply: We thank the reviewer for their comment. We have added the word "curate" to the sentence in Line 37.

2. The MAGs generated for this manuscript have been shared publicly on NCBI Bioproject, but no code for this analysis was listed. Are the tools used for this analysis all stand-alone applications, and thus no custom code was required?

Reply: We thank the reviewer for their comment. All tools used in this study were stand-alone applications; thus, no custom code was required. We have added the code used to perform all statistical analyses in the supplementary materials. The relevant code and data are hosted on GitHub, and the link was included as data availability (https://github.com/mdsufz/mockc_analysis).

Reviewer 3

Comment 1

In this work, de Rocha et al. test three different pipelines for extracting metagenome-assembled genomes (MAGs) from three metagenomic datasets while systematically varying the input parameters for the pipelines. Their aim was to evaluate how sample evenness, sequencing depth, and taxonomic distribution profiles influence the recovery of genomes. To accomplish this, they generated mock microbial communities by dividing 126 bacteria, archaea, and fungi into three groups of 42 each. They then tested three pipelines, 8K, DT, and MM, with sequencing depth, taxonomical distribution, and evenness distribution varied with five different parameters each. The results indicated that the 8Kpipeline recovered the highest number of MAGs but only 25% of the 42 species on average, while the DT pipeline recovered 45% of species on average. The 8K pipeline also recovered the lowest number of true positives and had the highest number of false negatives. Moreover, MAG recovery was influenced by taxonomic relatedness and sequencing depth but not evenness distribution. Overall, their results suggested that the DT pipeline has the highest accuracy and sequencing depth influences results. The three pipelines also had generally low accuracy in recovering closely related species. This is a timely study that demonstrates the importance of the choice of reconstruction pipeline and parameter selection. As MAG reconstruction is commonly used by the research community, this is valuable knowledge.

Reply 1: We thank the reviewer for their positive feedback.

Comment 2

While the MAGs have been made available, the code used to generate and evaluate the MAGs does not appear to be available. This is also important for the audience to reproduce how the quality and accuracy of the MAGs was determined.

Reply 2: We thank the reviewer for their comment. We have added the code used to perform all statistical analyses in the supplementary materials. The relevant code and data are hosted on GitHub, and the link was included as data availability (https://github.com/mdsufz/mockc_analysis). The tools used for the data generation are all stand-alone applications and do not require modified scripts.

Minor comments:

- Line 36: incomplete sentence "Our data indicates the scientific community should their findings from MAG recovery"

Reply: We thank the reviewer for their comment. We have added the word "curate" to the sentence in line 37.

- There are a few typos (e.g., "Informtaion" in line 50).

Reply: We thank the reviewer for their comment. We have corrected the typo.

- Line 104: other than in the abstract, this is the first time, the abbreviations for the three pipelines are used before being defined, which is a bit confusing.

Reply: We thank the reviewer for their comment. We have added the abbreviations to the main text in Lines 134-137 of the manuscript.

- It would be helpful to have the overview on the workflow near the beginning of the manuscript rather than as Figure 6.

Reply: We thank the reviewer for their comment. The methods section is placed by the journal at the end of the conclusion. However, we have added text in the manuscript in Lines 120-137 containing an overview of the workflow to improve the manuscript's clarity and readability. Additionally, we have completely restructured the results section to guide the reader through the work better.

- There is a high number of supplementary tables most of which are small. I would suggest condensing them.

Reply: We thank the reviewer for their comment. We agree that we have a high number of small supplementary tables. However, this was done intentionally to facilitate result visualization.
