# Supplementary material for: Simulation of 69 microbial communities indicates sequencing depth and false positives are major drivers of bias in prokaryotic metagenome-assembled genome recovery
Source: PLoS Comput Biol. 2024 Oct 22;20(10):e1012530. doi: 10.1371/journal.pcbi.1012530 (PMC11530072; doi:10.1371/journal.pcbi.1012530)
Supplement: Attachment — Submitted filename: 02_Second_PointbyPointReplytoReviewers_v8.pdf [file pcbi.1012530.s062.pdf]

Dear Editor,

We appreciate the comments and points raised by the editor and reviewers.

All comments have been considered or discussed in the updated version of our manuscript.

You will find the replies to all comments on the following pages. We indicated the different points made by the reviewers by “Comment X” and their answers by “Reply X.”

Sincerely,
Ulisses Rocha

Point by point Reply:

Editor:

Dear Dr Rocha,

Thank you very much for submitting your manuscript "Simulation of 69 microbial communities indicates sequencing depth and false positives are major drivers of bias in Prokaryotic metagenome-assembled genome recovery" for consideration at PLOS Computational Biology. As with all papers reviewed by the journal, your manuscript was reviewed by members of the editorial board and by several independent reviewers. The reviewers appreciated the attention to an important topic but raised important points that need to be further addressed.

Please prepare and submit your revised manuscript within 30 days. If you anticipate any delay, please let us know the expected resubmission date by replying to this email.

When you are ready to resubmit, please upload the following:

[1] A letter containing a detailed list of your responses to all review comments, and a description of the changes you have made in the manuscript. Please note while forming your response, if your article is accepted, you may have the opportunity to make the peer review history publicly available. The record will include editor decision letters (with reviews) and your responses to reviewer comments. If eligible, we will contact you to opt in or out

[2] Two versions of the revised manuscript: one with either highlights or tracked changes denoting where the text has been changed; the other a clean version (uploaded as the manuscript file).
Important additional instructions are given below your reviewer comments.

Thank you again for your submission to our journal. We hope that our editorial process has been constructive so far, and we welcome your feedback at any time. Please don't hesitate to contact us if you have any questions or comments.

Sincerely,

Jason Papin
Editor-in-Chief
PLOS Computational Biology

Reply to the editor:

Dear Papin,
Thank you for considering our manuscript and providing us with the reviewers' feedback. We have carefully addressed all the comments and have submitted a revised version of our manuscript, along with a detailed response below. We appreciate the constructive review process and look forward to your decision.

****

Reviewer #2:

Comment 1:
This manuscript evaluates several Metagenome-Assembled Genome pipelines and the effects of sequence depth, species abundance, and species relatedness on model performance. In this revision, the authors put substantial effort into revising the organization of the manuscript, and I found that the goals of the manuscript and major findings were much clearer and easier to follow. The authors also added a new Figure 1, which highlights the major differences across pipelines.

Reply 1: We thank you for the feedback on our revised manuscript and the work you put into carefully evaluating it. We address your other comments in detail below.

Comment 2:
Major Comments

1 – However, the statistical approach used in the manuscript is still a concern, as it underestimates the possibility of Type 1 errors in the results. Comments are listed below:
A Student's t test is used for nearly all of the analyses in the manuscript, despite most of these analyses comparing differences between 3 or more groups (the paper compared outcomes across 3 different MAG pipelines, 5 different sequence depths, and 4 different abundance models). While I understand that the pairwise comparisons between two groups is what you are interested in, using a t test across all comparisons will overestimate the significance of your results. This is because each comparison is associated with a Type 1 error, where you falsely reject the null hypothesis. The probability of a Type 1 error will increase with each comparison, and when you are performing 3 tests (i.e., 8K vs DT, 8K vs MM, DT vs MM) with the same data, using a p value threshold of $p < 0.05$ will underestimate this error rate. **The reported p values are also not corrected for multiple comparisons,** which occur when using a univariate test repeatedly across many comparisons. As is, the p values presented overestimate the number of statistical differences and must be adjusted to account for these issues.

A One-way ANOVA with a post hoc test (such as Tukey's method) will adjust the p value to account for the increased Type 1 error rate, and pairwise comparisons can be performed to identify statistical differences between paired conditions. For example, in Supplemental Table 6, a One-way ANOVA should be used to calculate pairwise comparisons across all Taxonomic Distributions for each Species Abundance. I would recommend this approach to account for the increased Type 1 error rate.

Reply 2: Thank you for your concern and attention. We appreciate your thorough review and comments regarding our statistical analysis. We acknowledge two oversights in our original manuscript: (1) Although the statistical differences were assigned using Bonferroni adjusted t-tests, we did not explicitly mention that we applied Bonferroni correction to our t-tests. The reviewer can verify that the Bonferroni adjustments were applied by checking the analysis files accompanying the manuscript, which are publicly available on Github (https://github.com/mdsufz/mockc_analysis/). More specifically, if the reviewer checks the file

"03_pairwise_analysis/00_mockc_pairwise_analysis.r" in line 38, line 108, and line 175 on the commit id f9865f7 from 7 months ago, they will see that the Bonferroni adjustment method was used. Second, (2) we oversaw the inclusion of the adjusted p-values in our supplementary tables. The Bonferroni correction was applied to all pairwise comparisons to account for multiple comparisons and control type 1 error. However, following your suggestion, we have also conducted One-way ANOVA with Tukey's post hoc test for all relevant comparisons. The new ANOVA and Tukey test results are available on the GitHub page associated with this manuscript, and the Bonferroni-adjusted p-values for all comparisons have been included in the relevant supplementary tables. Additionally, after careful comparison, we confirmed that both statistical approaches were consistent with our original findings, and no major changes to the discussion were necessary. We have included these changes in Lines 559 and 563-564.

After careful consideration and comparison of the results, we have decided to retain the Bonferroni-corrected t-tests in our primary analysis for two main reasons: 1) The Bonferroni correction provides a conservative approach in controlling Type I error, which aligns with our focus on metagenome-assembled genome (MAG) recovery analysis where we prioritize the reliability of true positives. Only comparisons in marginal cases were considered statistically different by the Tukey approach and not by the Bonferroni adjusted t-test, and we decided to follow with the more conservative approach; 2) Our research questions were primarily focused on specific pairwise comparisons between methods, which is well-suited to the t-test approach. It's important to note that our results and discussion were based on the statistical differences indicated by these Bonferroni-adjusted p-values.

We believe that the current discussion in the paper remains valid and relevant, as the conclusions drawn from the Bonferroni-corrected t-tests are supported by the additional ANOVA and Tukey's test results. The consistency between these methods reinforces the robustness of our findings. We appreciate your suggestion to use ANOVA with Tukey's post hoc test, and we have included these results alongside our original analysis to provide a comprehensive view of the data. Your valuable feedback has helped us improve the clarity and completeness of our statistical reporting.

Comment 3:
Minor Comments

2 – The resolution of the figures (particularly Figure 1 and Figure 4) appears low. Please check figure resolution before submission of final figures.

Reply 3: Thank you for your attention. We have applied the PACE digital diagnostic tool (https://pacev2.apexcovantage.com) from PLOS to all our figures. All the figures passed the validation procedure. We are re-uploading the figures downloaded from the PACE tool.

Reviewer #3

Comment 4

The authors have done a good job revising the manuscript and addressing my and the other reviewers' comments. I have no further comments.

Reply 4: We thank the reviewer for the attention and work.
